# Decoding the mechanisms underlying breast cancer brain metastasis: paving the way for precision therapeutics

**DOI:** 10.1186/s40364-025-00854-3

**Published:** 2025-11-11

**Authors:** Xuanhe Zhang, Xiang Wang, Saimeng Shi, Duancheng Guo

**Affiliations:** 1 Department of Radiology, First People’s Hospital of Linping District, Linping Campus, The Second Affiliated Hospital of Zhejiang University School of Medicine, Hangzhou, 311100 China; 2 Cancer Institute, Fudan University Shanghai Cancer Center, Shanghai, 200032 China; 3 Department of Oncology, Shanghai Medical College, Fudan University, Shanghai, 200032 China; 4 State Key Laboratory of Bioreactor Engineering, Shanghai Key Laboratory of New Drug Design, and School of Pharmacy, East China University of Science and Technology, Shanghai, 200237 China

**Keywords:** Breast cancer, Brain metastasis, Tumor microenvironment, Clinical therapy

## Abstract

The development of brain metastasis is a major cause of significantly reduced survival in breast cancer patients. The initiation and progression of breast cancer brain metastasis (BCBM) involve multiple distinct molecular pathways and reprogramming of the tumor microenvironment (TME). This review systematically summarizes key mechanisms underlying BCBM, including epithelial-mesenchymal transition (EMT), extracellular matrix (ECM) remodeling, and the spatiotemporal dynamics of metabolic reprogramming regulated by critical signaling pathways during brain colonization. In particular, we highlight emerging mechanisms of breaching the specialized brain multifunctional barriers. Furthermore, this review provides an in-depth analysis of the cooperative immune-suppressive network within the BCBM TME, emphasizing the crosstalk among various immune cell components (such as T cells, B cells, macrophages, neutrophils, NK cells, MDSCs) and intracranial-specific cellular elements (including astrocytes, microglia, brain metastasis-associated fibroblasts). Through the complex interplay, these cells collectively facilitate immune evasion and metastatic outgrowth. Accordingly, we discuss the current clinical management of BCBM and potential future directions. Deeper mechanistic insights are expected to offer novel biomarkers and reveal new targets for developing precision therapeutic strategies against BCBM.

## Graphical abstract

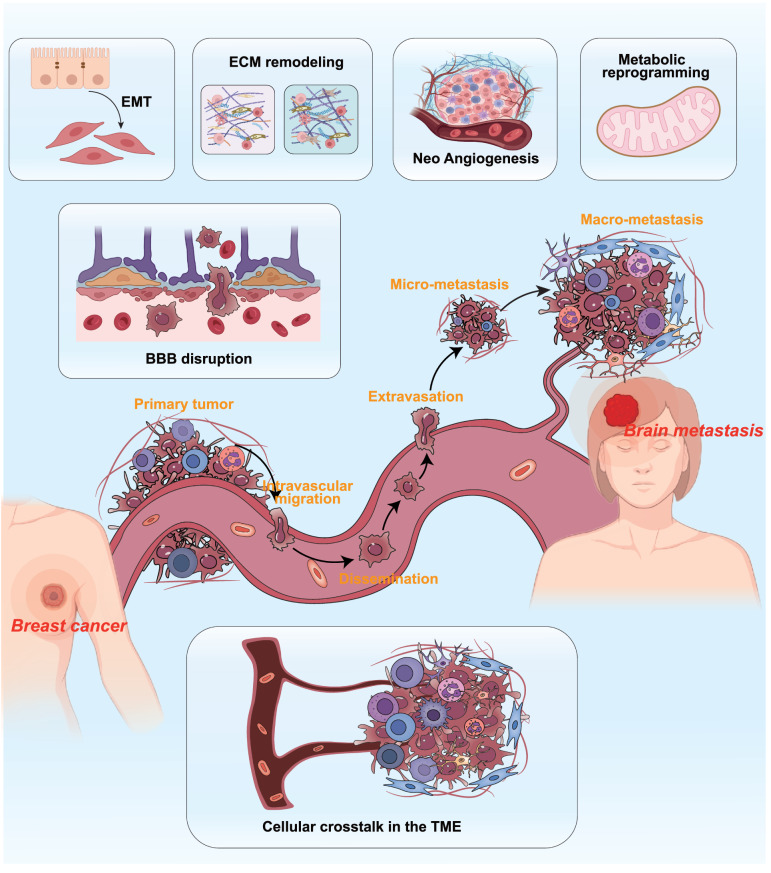

## Introduction

Breast cancer ranks as the most commonly diagnosed cancer worldwide [1, 2]. In recent decades, as the survival rates for breast cancer patients continue to rise, so too does the occurrence of distant metastasis. Breast cancer cells show a tendency toward specific metastatic sites, with the brain being one of the common locations for breast cancer metastasis. Studies have indicated that approximately 15–25% of breast cancer patients develop brain metastasis [3, 4]. Specifically, this includes around 30% of patients with human epidermal growth factor receptor 2 (HER2)-positive breast cancer (HER2BC, an overexpression of HER2), 30–50% of those with triple-negative breast cancer (TNBC, lacking expression of estrogen receptor (ER), progesterone receptor (PR), and HER2), and 15% of patients with hormone receptors (HR)-positive breast cancer (HRBC, an overexpression of ER or PR) [5, 6]. The development of brain metastasis in breast cancer patients is significantly associated with reduced survival rates and a decline in the quality of life. Indeed, when breast cancer metastasizes to the central nervous system (CNS), the median overall survival (OS) is dramatically reduced to about 7 months, and the one-year survival rate drops to a mere 20% [7, 8].

The fundamental cause of breast cancer brain metastasis (BCBM) lies in highly malignant subclones of breast cancer cells that, during systemic dissemination, successfully breach the tightly guarded and highly selective blood-brain barrier (BBB), a key feature distinguishing brain metastasis from metastasis to other sites [9–11]. Furthermore, they develop strategies to survive, adapt, proliferate, and establish blood supply within the brain’s unique microenvironment, which is inherently inhospitable to the survival of foreign cells. Key risk factors include specific molecular subtypes of breast cancer, such as HER2BC and TNBC, late-stage diagnosis, previous treatment history, nodal stages, high tumor burden and younger age at initial diagnosis [12–14]. The brain possesses a unique microenvironment, characterized by highly specialized resident cells and distinct neurogenic niches, which exerts strong selective pressure on circulating disseminated breast cancer cells. Successful intracranial metastatic colonization is a bidirectional process wherein cancer cells adapt to the brain’s unique microenvironment and coordinately reshape the brain metastatic niche. Disseminated breast cancer cells achieve successful penetration of multiple functional barriers and engage in crosstalk with intracranial immune cell components (e.g., T cells, B cells, macrophages, neutrophils, NK cells, MDSCs) as well as brain-specific cellular components (e.g., astrocytes, microglia, brain metastasis-associated fibroblasts). The cumulative effects of these interactions ultimately drive the development of brain metastasis of breast cancer. Once brain metastasis occurs, the presence of the blood-brain barrier (BBB) serves as a “sanctuary” for cancer cells. Most therapeutic agents struggle to penetrate the BBB and reach the brain, thereby limiting the efficacy of brain tumor treatments [15, 16].

Currently, the standard first-line treatment regimens for breast cancer are primarily driven according to the molecular subtypes. Patients with the HER2BC routinely receive anti-HER2 monoclonal or dual antibodies combined with chemotherapy; those with the HR+/HER2- subtype are treated upfront with CDK4/6i plus endocrine therapy; while patients with TNBC typically receive chemotherapy with or without immunotherapy [9, 17, 18]. Building on this foundation, the development of breakthrough agents has continued to advance. For instance, the advent of next-generation selective ER degraders, such as elacestrant, antibody-drug conjugates (ADCs) such as trastuzumab deruxtecan (T-DXd) and trastuzumab emtansine (T-DM1), Poly (ADP-ribose) polymerase (PARP) inhibitors, greatly improving patient survival benefits. Continuous breakthroughs have been achieved in the early detection, therapeutic approaches and the exploration of underlying mechanisms of primary breast cancer, all of which have contributed to a steady decline in the overall mortality rate of breast cancer [19]. Despite these progresses, the clinical outlook for patients with BCBM remains extremely grim. The presence of the BBB restricts drug entry into the brain. Molecule tyrosine kinase inhibitors (TKIs) such as tucatinib, neratinib, and lapatinib, owing to their small molecular weight and favorable lipophilicity, are relatively more capable of penetrating the BBB [20]. ADCs like T-DXd have also demonstrated durable intracranial activity in multiple clinical studies, benefiting patients with baseline brain metastasis or those who develop brain metastasis during treatment [21–23]. However, the efficiency of drugs that truly penetrate the BBB, reach the effective regions of brain parenchyma, and achieve sufficient therapeutic concentrations remains limited. Additionally, the presence of efflux pumps (e.g., P-gp, BCRP) further results in insufficient effective drug concentrations and the development of drug resistance [24]. Drug delivery systems such as nanoparticles and focused ultrasound for BBB opening are still immature, which remain in the exploratory stage and have limited clinical application [25–27].

The treatment of BCBM faces severe practical challenges. The key reason lies in the fact that the underlying mechanisms of brain metastasis remain not fully understood, and how the brain’s unique microenvironment promotes the development of metastasis is crucial for identifying potential therapeutic targets and effective treatment strategies. This review discusses the unique mechanisms involved in the process of BCBM, with a particular focus on the distinctive tumor microenvironment (TME) of brain metastasis. Additionally, it addresses the current clinical applications in the diagnosis and treatment of BCBM, as well as potential future research directions. Our work aims to provide a systematic and comprehensive understanding for BCBM, which will facilitate the identification of effective therapeutic targets and the development of more efficacious treatment regimens.

## Fundamental process of breast cancer metastasizing to the brain

The occurrence and development of metastasis is a dynamic, multifaceted, and multi-step process, which fundamentally encompasses the following phases: (1) local invasion: cancer cells detach from the original tumor mass and infiltrate the adjacent tissues; (2) intravascular migration: cancer cells gain entry into the bloodstream or lymphatic vessels; (3) dissemination: cancer cells are transported to other regions of the body via the circulatory or lymphatic systems; (4) extravasation: cancer cells exit the circulation and infiltrate distant organs and tissues; (5) micro-metastasis and dormancy: cancer cells multiply at the new location, initiating the formation of micro-metastases, and the majority of these micro-metastatic cancer clusters enter a state of dormancy; (6) pre-metastatic niche and macro-metastasis: as micro-metastatic tumors overcome dormancy and growth suppression, they resume growth at secondary locations, develop a pre-metastatic niche, and progressively evolve into sizable metastatic tumors or additional metastases [28–30]. These steps may occur overlapped in time. During the whole process, a cascade of biological events unfolds, endowing cancer cells with the capacity to journey from the primary tumor to a distant organ, where they can take root and establish themselves [31, 32].

**Fig. 1:**
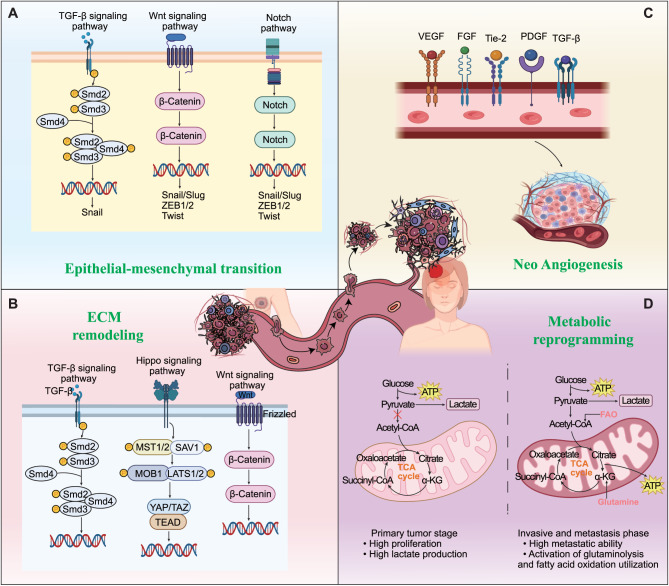
Significant biological events and their associated mechanisms during the cascade process of breast cancer brain metastasis. (**A**) The induction of epithelial-mesenchymal transition (EMT) in breast cancer is predominantly mediated by the activation of several crucial signaling pathways, namely the transforming growth factor-β (TGF-β), Wnt, and Notch signaling pathways. The TGF - β signaling pathway triggers the activation of snail, whereas the Notch and Wnt signaling pathways lead to the activation of Snail, Twist, and Zeb. These activations collectively drive the progression of EMT. (**B**) Crucial signaling pathways governing extracellular matrix (ECM) remodeling during breast cancer brain metastasis (BCBM) encompass the transforming growth factor-β (TGF-β) signaling pathway, the Wnt signaling cascade, and the Hippo signaling axis. (**C**) Members of the vascular endothelial growth factor (VEGF) family, the angiopoietin-Tie2 pathway, fibroblast growth factor (FGF), platelet-derived growth factor (PDGF), and transforming growth factor-β (TGF-β) are key regulators in the dynamic process of angiogenesis in breast cancer, which is indispensable for tumor growth, invasion, and metastasis. (**D**) Metabolic reprogramming in cancer cells from the primary tumor stage to the metastatic stage

A series of biological events takes place, conferring on cancer cells the capability to migrate from the original tumor site to a distant organ, where they can implant and establish a foothold. Epithelial–mesenchymal transition (EMT) is recognized as a pivotal process in the initiation of BCBM [32–35]. Multiple signaling pathways are known to drive EMT, with the TGF-β/SMAD–SNAIL axis, Wnt/β-catenin, and Notch being the most extensively studied. TGF-β signaling activates Snail, while Notch and Wnt signaling promote the expression of Snail, Twist, and Zeb, collectively facilitating EMT (Fig. 1A) [36–38]. Various transcription factors, microRNAs, and pharmacological agents may modulate EMT by activating or inhibiting these pathways (Table 1). Notably, although multiple pathways can induce EMT, their relative predominance in the context of BCBM is contingent upon tumor subtype and the experimental model employed. A synthesis of single-cell and spatial omics data and clinical evidence revealed significant disparities in the intracerebral tumor architecture and microenvironment between HER2BC and TNBC, which are the two subtypes most predisposed to cerebral metastasis [10]. This heterogeneity likely reflects a differential reliance on distinct EMT-driving axes. Substantial evidence indicates that HER2+ BCBM is predominantly governed by the TGF-β/SMAD-SNAIL axis, whereas TNBC more frequently exhibits characteristics indicative of Wnt/β-catenin pathway activation [39, 78, 79]. This finding implicates the existence of subtype-specific therapeutic vulnerabilities and suggests that distinct priority targets may exist for these respective cohorts. Targeted modulation of the aforementioned pathways, through drugs, inhibitors, or activators, to suppress the initiation and progression of BCBM may represent a promising therapeutic strategy.

**Table 1:** Key signaling pathways and molecules involved in the epithelial-mesenchymal transition of breast cancer brain metastasis

Signaling pathways	Regulatory molecules/factors/drugs	References
Wnt signaling pathways	Activating: PROX1, Aquaporin 5, SLC35A2, Nectin-4, HMGA1, INHBA, XB130, DNER, LINC00518, MiR-125b, MiR-374a	[39–49]
	Inhibiting: miR-29, lncRNA AK023507	[50, 51]
	Drugs/compounds: flavonoid baicalein, astragalus polysaccharide, apatinib, pyrvinium pamoate, jatrophone, FL118, TIMP-2	[37, 52–57]
Notch signaling pathways	Activating: COX-2, hypoxia, SCUBE2	[58–60]
	Inhibiting: NUMB, Notch3, GSK3β	[61, 62]
	Drugs/compounds: cimigenoside, 3,6-dihydroxyflavone, MHP-1	[63–65]
TGF-β signaling pathways	Activating: ICAM1, FUT8, heat treatment-induced autophagy, cPLA2α, CdGAP, MiR-487a	[66–71]
	Inhibiting: miR-190, miR-133b	[72, 73]
	Drugs/compounds: calycosin, Xihuang pill, WSZG, Pirfenidone	[74–77]

Extracellular matrix (ECM) remodeling is a key driver of local invasion and intravascular dissemination in BCBM. Signaling pathways such as TGF-β, Wnt/β-catenin, and Hippo regulate ECM-modifying enzymes, such as MMPs, heparanases and LOX, as well as ECM proteins, thereby altering the mechanical and structural properties of the microenvironment and facilitating BBB disruption and tumor cell colonization in the brain [80–82]. Figure 1B illustrates the pivotal signaling pathways associated with ECM-related events during BCBM.

Angiogenesis is a crucial component of tumor metastasis as well [83, 84]. During the initial phase of metastasis, newly formed abnormal and proliferative blood vessels serve as vital pathways for tumor cells to flee from the primary location. Upon reaching distant sites, the new tumor tissue prompts angiogenesis to supply the nutrients and oxygen essential for supporting extensive metastases. Breast cancer angiogenesis is a complex biological process that involves numerous signaling pathways and molecular components. The vascular endothelial growth factor (VEGF) pathway stands as the central driving force behind angiogenesis. VEGF is a family of growth factors, encompassing VEGF-A, VEGF-B, VEGF-C, and others, with VEGF-A being the predominant member [85]. This factor plays a pivotal role in promoting the growth and development of new blood vessels. The angiopoietin-Tie2 pathway functions as a regulator of vascular stability [86]. Angiopoietin-1 (Ang-1) and angiopoietin-2 (Ang-2) exert their effects by binding to the Tie2 receptor present on endothelial cells. Ang-1 promotes vessel maturation and stability, while Ang-2 can disrupt the quiescent state of blood vessels, facilitating angiogenesis under certain conditions [87, 88]. Moreover, several other factors, including fibroblast growth factor (FGF), platelet-derived growth factor (PDGF), and transforming growth factor-β (TGF-β), are also crucial for angiogenesis [89]. These factors interact intricately with each other and with the aforementioned pathways, contributing to the dynamic process of new blood vessel formation in breast cancer, which is essential for tumor growth, invasion, and metastasis (Fig. 1C).

Cancer cells also demonstrate remarkable metabolic plasticity during metastasis, dynamically adapting their energy metabolism to meet the demands of diverse microenvironments [90]. This adaptability is crucial for successful metastatic transition. During the primary tumor stage, proliferative demands drive Warburg effect dominance, where cancer cells preferentially utilize glycolysis over mitochondrial oxidative phosphorylation for glucose processing, even under normoxic conditions. This glycolytic flux not only supports rapid biomass generation but also acidifies the TME through lactate secretion, facilitating immune evasion and initial invasion [91, 92]. As cells transition to the invasive and metastasis phase, metabolic priorities shift toward energy-intensive processes. Enhanced tricarboxylic acid (TCA) cycle activity elevates ATP production to power cytoskeletal remodeling and motility machinery [93, 94]. When TCA intermediates become limiting, glutaminolysis is activated, with glutamine serving as an alternative fuel source to replenish cycle intermediates and sustain ATP generation [95]. Concurrently, lipid metabolic reprogramming provides essential membrane components and signaling molecules that promote invasive phenotypes. Upon entering the pre-metastatic niche, cancer cells adapt their metabolism to the local environment. Distinct metastatic sites induce organ-specific metabolic adaptations. For example, brain metastases demonstrate preferential fatty acid oxidation utilization, while osseous lesions exploit lactate-rich environments through lactate dehydrogenase-mediated metabolic coupling [95]. This spatiotemporal metabolic rewiring enables metastatic cells to satisfy divergent bioenergetic demands while simultaneously remodeling target niches to favor colonization. Throughout the metastatic cascade, this dynamic metabolic reprogramming serves dual purposes: supporting immediate survival/proliferation needs while preconditioning distal sites through metabolite-mediated niche modification (Fig. 1D).

Based on existing studies, breast cancer metastasis is not driven by a single pathway, but rather results from a dynamic coupling of EMT, ECM remodeling, angiogenesis, and metabolic reprogramming. Variations in molecular subtypes and experimental models may contribute to inconsistencies in research findings. Moving forward, prioritizing mechanisms according to subtype specificity and emphasizing combination strategies, rather than targeting isolated pathways, may facilitate the development of more effective translational therapies. Various types of tumors exhibit distinct preferences when it comes to choosing metastatic destinations. For instance, breast cancer often metastasizes to the bones, lungs, and brain, while colorectal and pancreatic cancers frequently spread to the liver, and ovarian tumors often lead to metastases in the abdomen [96–99]. In 1889, Stephen Paget proposed the “seed and soil” hypothesis, which was widely accepted [100]. This hypothesis holds that particular tumor cells (the seeds) are inclined to travel to particular organs (the soil), and that the appropriate soil environment is beneficial for the seeds to take root and grow, effectively accounting for the preferential sites of metastasis. Brain metastases are commonly observed in patients suffering from breast cancer, lung cancer, and melanoma [101]. The brain metastatic site features a distinct TME, with unique cell types, special environment and metabolic characteristics, all of which impose a specific selective pressure on cancer cells. Consequently, an in-depth investigation into the TME of BCBM could aid in comprehending the mechanisms behind such metastases and in identifying novel therapeutic targets and treatment strategies.

## Multifunctional barrier and brain metastasis

**Fig. 2:**
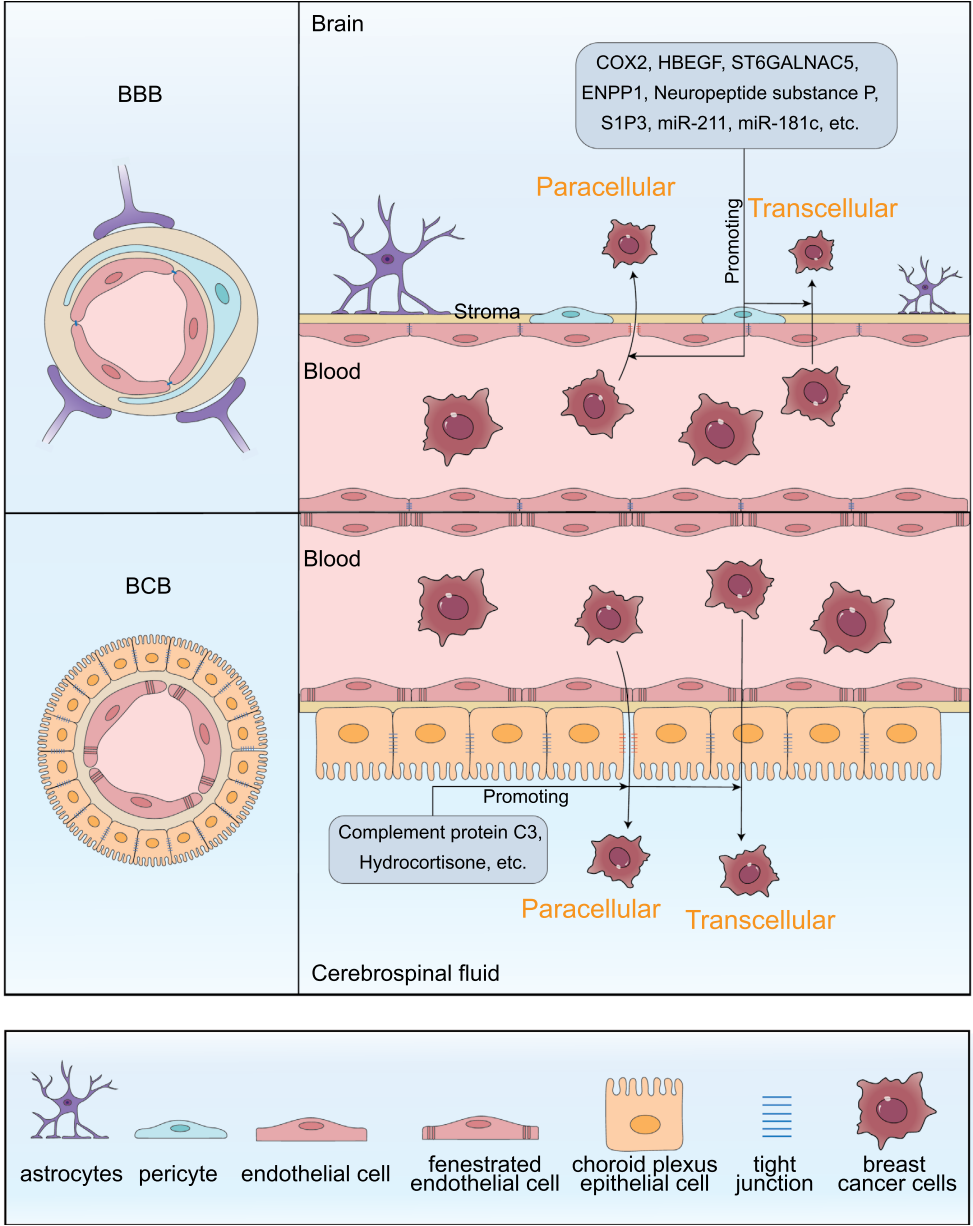
Multifunctional barriers of the brain: the blood-brain barrier (BBB) and the blood-cerebrospinal fluid barrier (BCFB). The BBB and the BCFB are two essential multifunctional barriers that maintain the homeostasis and integrity of the central nervous system (CNS). The BBB serves as a dynamic interface between the brain’s vascular system and the nervous tissue, primarily composed of three critical structures. The innermost layer consists of brain microvascular endothelial cells (BMECs) that are interconnected by tight junctions, creating the capillary wall. On the exterior side of the endothelial cells are pericytes, which produce a substantial amount of extracellular matrix along with BMECs, forming a basal membrane and enveloping themselves. The outermost structure is constituted by the end feet of astrocytes that project outwards. The BCFB is located in the choroid plexus and is constituted by choroidal epithelial cells. These cells are connected by tight junctions and are attached to the underlying blood vessels via a basal lamina. The choroidal capillaries are highly fenestrated. Metastatic tumor cells can transmigrate the multifunctional barriers via the paracellular pathway and the transcellular pathway. A variety of substances have been identified as potential modulators of barrier permeability in the context of brain metastasis. Molecules such as COX2, HBEGF, ST6GALNAC5, ENPP1, neuropeptide substance P, S1P3, miR-211, and miR-181c may enhance the permeability of the BBB, thereby facilitating the transmigration of cancer cells across this barrier. similarly, molecules like complement protein C3 and hydrocortisone may play a role in promoting cancer cell traversal across the BCFB

The BBB and the blood-cerebrospinal fluid barrier (BCFB) are core structures responsible for maintaining homeostasis and security within the CNS, and they constitute the first line of defense against tumor cell invasion into the brain [102] (Fig. 2). Metastatic tumor cells can transmigrate the BBB via two distinct routes: the paracellular pathway, which involves passing through tight junctions between endothelial cells, and the transcellular pathway, whereby cells traverse through the endothelial cells themselves [103]. Concurrently, disruption of the BCFB is closely associated with the development of ventricular and meningeal metastasis.

Multiple factors have been shown to compromise the integrity of the BBB or BCFB (Table 2). For instance, preclinical evidence indicates that ENPP1, secreted from primary breast cancer, can induce endothelial cell dysfunction by impairing insulin signaling and its downstream AKT/GSK3β/β-catenin pathway [106]. This disruption leads to the compromise of BBB integrity with a loss of tight and adherens junction proteins. Circular RNA, designated as circBCBM1 (circ_0001944), can upregulate the expression of BRD4 by suppressing the activity of miR-125a [118]. This leads to an upregulation of matrix metallopeptidase 9 (MMP9) expression through the activation of the Sonic Hedgehog (SHH) signaling pathway. The overexpression and secretion of MMP9 enhance the permeability of the BBB, thereby facilitating the transendothelial migration of breast cancer cells and promoting the BCBM. Various tumor cells, including breast cancer, can secrete the complement protein C3. This secretion activates the C3a receptor within the epithelium of the choroid plexus, which in turn leads to the destruction of the BCFB [116]. The compromised BCFB facilitates the occurrence of meningeal metastasis. Furthermore, extracellular vesicles derived from tumor cells have been identified to compromise the intact BBB by affecting the expression of rab7 in brain endothelial cells [119].

**Table 2:** Key molecules and mechanisms modulate the permeability and integrity of the multifunctional barrier

Molecule	Mechanism	Consequences	Reference
COX2, HBEGF	COX2 and HBEGF enhances the ability of cancer cells to extravasate through the non-fenestrated capillaries of the brain.	Compromising the integrity of the blood-brain barrier (BBB) and facilitating cancer cells to cross the BBB.	[104, 105]
ST6GALNAC5	ST6GALNAC5 expression is co-selected with, and acts as a specific mediator of, cancer cell infiltration through the BBB.	Compromising the integrity of the BBB and facilitating cancer cells to cross the BBB.	[104]
ENPP1	ENPP1 induces endothelial cell dysfunction by impairing insulin signaling and its downstream AKT/GSK3β/β-catenin pathway.	ENPP1 compromises the integrity of the BBB, with the loss of tight and adherens junction proteins, facilitating cancer cells to cross the BBB.	[106]
miR-211	miR-211 enhances the trans-BBB permeability, BBB adhesion, and stemness properties of breast cancer cells via the miR-211-SOX11–NGN2 axis.	Compromising the integrity of the BBB and facilitating cancer cells to cross the BBB.	[107]
lncRNA GS1-600G8.5	The lncRNA GS1-600G8.5 disrupts the integrity and increases the permeability of the BBB, and promotes the traversal of breast cancer cells across the endothelial cells. monolayer.	Compromising the integrity of the BBB and facilitating cancer cells to cross the BBB.	[108]
VGF (nerve growth factor inducible)	VGF induces substantial disruption of the BBB.	Compromising the integrity of the BBB and facilitating cancer cells to cross the BBB.	[109]
αB-crystallin	αB-crystallin enhances the adhesion of breast cancer cells to human microvascular endothelial cells, transendothelial migration, and BBB transmigration via an α3β1 integrin-dependent mechanism.	Facilitating cancer cells to cross the BBB.	[110]
ENT2	ENT2 facilitates the brain endothelial cell penetration and blood-brain barrier transport of a tumor-targeting anti-DNA autoantibody.	It is anticipated to significantly advance the field of brain tumor immunotherapy.	[111]
Neuropeptide substance P	Neuropeptide substance P stimulates human brain microvascular endothelial cells to secrete TNF-α and Ang-2. This leads to alterations in the localization and distribution of tight junction proteins, specifically zonula occludens-1 and claudin-5, and consequently increases the permeability of endothelial cells.	BBB damage and breast cancer cell colonization in the brain.	[112]
miR-181c	miR-181c promotes the disruption of the BBB through abnormal actin localization, by downregulating its target gene PDPK1.	BBB damage and breast cancer cell crossing to the brain.	[113]
Astrocytic sphingosine-1 phosphate receptor 3 (S1P3)	S1P3 signaling induces the secretion of IL-6 and CCL2 in astrocytes, which enhances the permeability of the functional brain barrier, thereby weakening endothelial cell adhesion.	Increased permeability of the multifunctional barrier.	[114]
Tumor cell-derived extracellular vesicles	Tumor-derived extracellular vesicles induce alterations in the cytoplasmic and cytoskeletal organization and dynamics of brain endothelial cells, thereby causing changes in cell morphology and motility.	Compromising the integrity of the BBB and facilitating cancer cells to cross the BBB.	[115]
Complement protein C3	Cancer cell-derived C3 activates the C3a receptor in the choroid plexus epithelium, thereby disrupting the blood–cerebrospinal fluid barrier.	Plasma components enter the cerebrospinal fluid and promote cancer cell growth.	[116]
Hydrocortisone	Hydrocortisone enhances the tightness of the blood-cerebrospinal fluid barrier (BCB) by upregulating the tight junction protein claudin-5; Hydrocortisone increases metastatic breast cancer cell transmigration across the BCB.	Increased cortisol levels facilitate breast-to-brain metastasis through the BCFB.	[117]

In summary, the mechanisms by which breast cancer cells traverse the BBB or BCFB involve disruption of tight junctions (e.g., via ENPP1 and MMP-9), regulation of signaling pathways (such as the circBCBM1–BRD4–SHH axis), immune-mediated processes (complement C3–C3aR), and exosome-driven transcellular transport. These mechanistic insights not only deepen our understanding of the early stages of brain metastasis but also offer potential avenues for clinical intervention. On one hand, molecules associated with barrier disruption could serve as biomarkers for identifying high-risk patients, enabling early risk stratification and dynamic monitoring. On the other hand, exosome-mediated trans-barrier signaling suggests that inhibiting specific exosomal cargo or blocking their uptake by endothelial cells may help delay metastatic progression. Furthermore, BCFB-specific mechanisms provide novel targets for precise intervention in meningeal metastasis. In Table 2, we catalog molecules with confirmed potential to disrupt the integrity of the BBB and BCFB. These molecules represent promising candidates for future research into biomarkers and targeted therapeutic strategies. Besides, the process of barrier disruption may also be leveraged therapeutically in a “reverse” manner. Limiting barrier damage could reduce metastatic seeding, while controlled and transient modulation of barrier function may enhance CNS delivery of antitumor drugs. In the future, key directions for enhancing the therapeutic effect of BCBM may include instantaneously opening multifunctional barriers through physical means, nanomedicines and intelligent delivery systems, as well as the synergy between barrier repair and drug delivery.

## The TME of BCBM

As emerging technologies such as single-cell RNA sequencing, multi-label immunofluorescence, multi-molecular imaging, transcriptome sequencing, mass spectrometry flow cytometry, microfluidics and organoid technology, and spatial analysis gain traction in oncology, researchers are gaining profound insights into the diverse components of the TME and their respective roles. The TME of brain metastases is a sophisticated ecosystem. The TME at the primary site is mainly composed of tumor cells, immune cells, stromal cells and extracellular matrix, while the brain metastasis has a more complex ecological niche, and only metastases adapted to this unique ecology can form intracranial metastases. Different from the cellular composition of the primary tumor, the brain itself possesses some unique cellular components, including neurons, astrocytes, and microglia, which are absent in the extracranial organs, thus forming a unique intracranial niche. Furthermore, the presence and constraints of the BBB play a significant role in filtering out most metastatic cells during the early stages of their invasion into the brain. The existence of the BBB also imparts a unique immune microenvironment of the brain, because the activity of immune cells to penetrate the BBB into the intracranial is limited as well. Further study of the TME in BCBM may enhance our comprehension of the processes and mechanisms underlying brain metastasis in breast cancer. In the following section, we would discuss the major cell types in the BCBM microenvironment and the roles they play.

### Cancer cells in the TME of BCBM

The process of metastasis initiates with the migration of cancer cells from their primary site. The “cellular plasticity” model posits that cells, under the influence of both intrinsic and extrinsic factors, are capable of reprogramming and altering their destiny and identity [120]. Throughout the trajectory of cancer progression, cellular plasticity endows cancer cells with the versatility to transition between diverse cellular states.

As previously discussed, upon detaching from the primary tumor, breast cancer cells are able to overcome selective pressures and facilitate their own metastasis by enhancing their stemness properties, undergoing EMT, and engaging in metabolic remodeling, among other mechanisms. The metastatic process encompasses a sequential series of steps, in which breast cancer cells first invade the surrounding tissues by breaching the basement membrane, then infiltrate into blood vessels. Subsequently, they manage to survive and circulate within the bloodstream, extravasate through transendothelial migration, and ultimately penetrate into the brain tissue by taking advantage of the increased permeability of the BBB.

Owing to the body’s potent anti-tumor capacity, a substantial proportion of metastatic cells undergo cell death, while the vast majority of the surviving cells enter a dormant state. Dormancy is considered an outcome of an adaptive response, during which cancer cells temporarily halt their proliferation and elude the stress imposed by the microenvironment. In breast cancer, the activation of the p38 signaling pathway inhibits the activity of extracellular signal-regulated kinase (ERK), a process that may trigger metastatic dormancy [121, 122]. Biglycan and reduced glycolysis are also implicated in the dormancy of breast cancer cells within the brain [123]. Conversely, increased concentrations of hormones, including estradiol and progesterone, can facilitate the reactivation of dormant breast cancer cells [124, 125]. Recent investigations have increasingly turned their attention to the regulatory function of immune cells in the dormancy of breast cancer cells. Studies have established that laminin 211 (LN211) secreted by astrocytes can trigger the dormancy of breast cancer cells. The underlying mechanism involves the induction of an interaction between dystroglycan receptor and yes-associated protein (YAP) [126]. Natural killer (NK) cells play a crucial role in maintaining the dormant state of breast cancer cells through the production of interferon-gamma (IFN-γ) [127, 128]. When breast cancer metastasizes to the lungs, alveolar macrophages express TGF-β2. Through the continuous interaction between macrophages and cancer cells mediated by the TGF-βRIII receptor, cancer cells are kept in a dormant state [129]. Conversely, the depletion of TGF-β2 triggers the reactivation of metastasis.

**Fig. 3:**
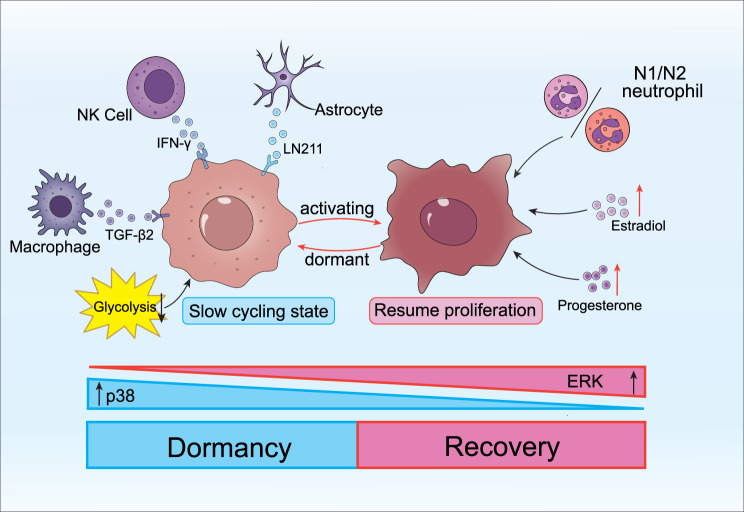
The dormancy and reactivation of cancer cells during the brain metastasis of breast cancer. After breast cancer cells metastasize to the brain, they enter a dormant state and halt proliferation under the combined influence of endogenous mechanisms, such as the p38 signaling pathway and the activity of extracellular signal-regulated kinase (ERK), as well as exogenous factors, including environmental cues and the functions of diverse immune cells. This dormancy serves as a survival strategy to evade the selective pressures imposed by the brain microenvironment. Following an extended period of quiescence, when suitable conditions are met, these dormant breast cancer cells can be reactivated and resume the cell cycle, thereby initiating massive metastasis in the brain

After an extended period of quiescence, dormant breast cancer cells can be activated and re-enter the cell cycle (Fig. 3). This reactivation occurs when suitable conditions arise, triggered by the coordinated action of both intrinsic and extrinsic mechanisms. These cancer cells engage in intricate crosstalk with various elements within the intracranial TME. By leveraging the synergistic effects of multiple pathways, they manage to evade immune surveillance, ultimately succeeding in establishing brain colonization and massive metastasis.

### Adaptive immune cells in the TME of BCBM

**Fig. 4:**
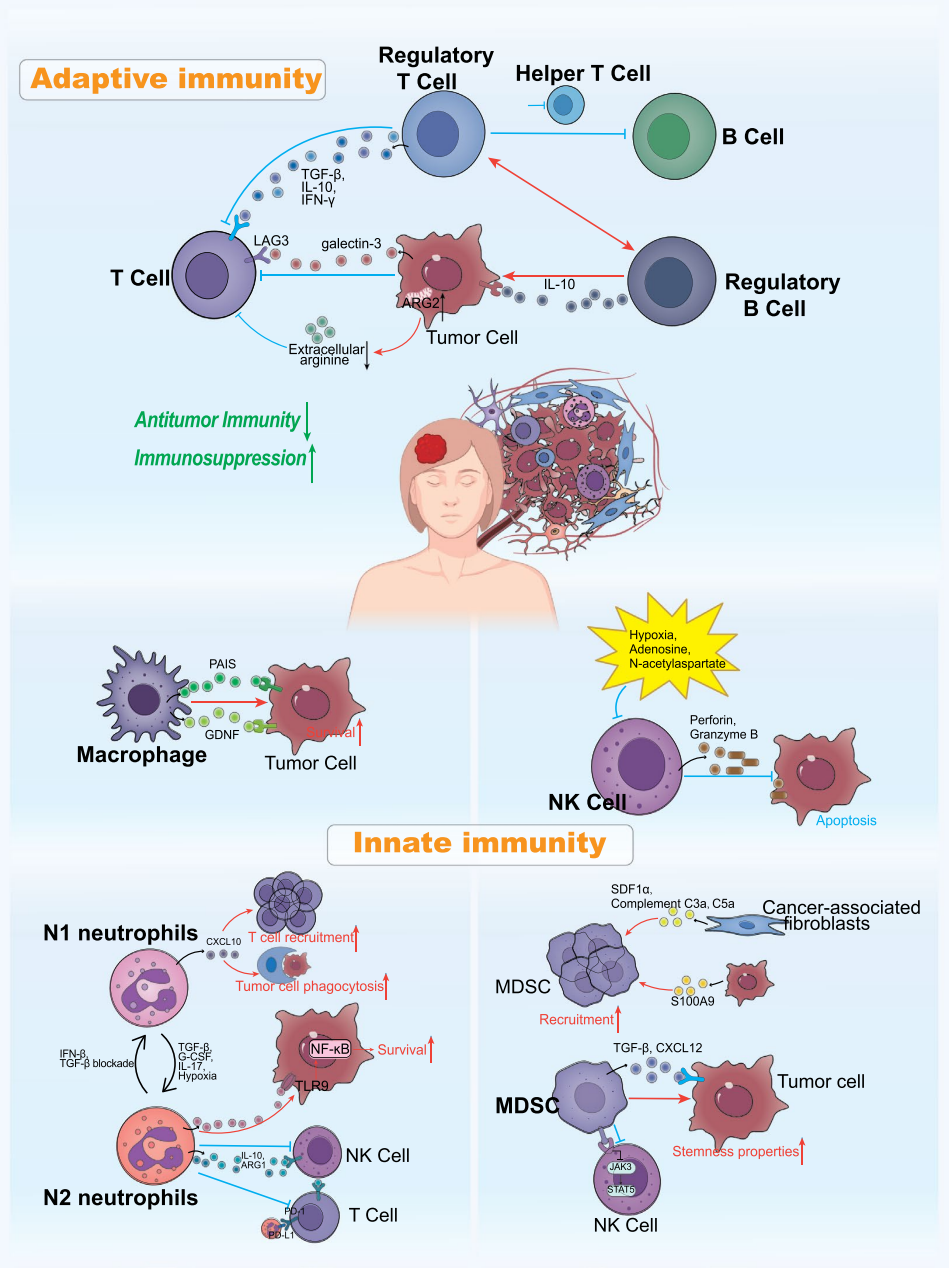
The immune microenvironment of breast cancer brain metastases: Adaptive immunity and innate immunity. The tumor microenvironment of brain metastases represents a sophisticated and intricate ecosystem. Brain metastases are not merely composed of tumor cells; Rather, they are populated by a diverse array of innate and adaptive immune cells. Through the mediation of various signaling molecules, tumor cells and different immune cell components engage in a complex interplay of cross-interference, ultimately giving rise to a distinctive immune microenvironment specific to breast cancer brain metastases

The profound immunosuppression observed in BCBM is not coincidental. Rather, it is actively orchestrated through interactions between tumor cells and the microenvironment. A hallmark of this process is the dysfunction of adaptive immunity (Fig. 4). CD8^+^ T cells exhibit weakened function and reduced infiltration, while regulatory T cells (Tregs) dominate, contributing to immunosuppression. Zou et al. mapped a high-resolution tumor ecosystem for liver and brain metastases in breast cancer through single-cell RNA sequencing of 44473 cells [130]. Their studies revealed significant reprogramming of immunosuppressive cells within the metastatic microenvironment, including FOXP3^+^ Tregs, LAMP3^+^ tolerogenic dendritic cells, and CCL18^+^ M2-type macrophages, compared to primary tumors. The galectin-3 produced by tumor cells and macrophages blocks CD8^+^ T cell activation signals and induces T cell apoptosis by interacting with LAG3 on the T cell surface; while FOXP3^+^ Tregs suppress anti-tumor immunity by secreting various inhibitory cytokines, such as TGF-β, IL-10 and IFN-γ. Furthermore, the expression level of the immunosuppressive gene ARG2 was upregulated in tumor cells and myeloid-derived cells. ARG2 plays a crucial role in orchestrating the immunosuppressive microenvironment by inducing the depletion of extracellular arginine, thereby impeding the proliferation of CD8^+^ T cells [131].

It is noteworthy that this immunosuppressive network exhibits a high degree of synergy. For instance, Tregs not only directly suppress effector T cells but may also limit B cell antibody production by inhibiting the function of follicular helper T cells [132]. Additionally, studies have shown that Tregs can promote the expansion of IL-10-producing B cells (immunosuppressive B cells, also known as Bregs) in breast cancer [133]. In turn, the increased presence of IL-10-secreting Bregs enhances Treg function [134]. Tumor-infiltrating B cells can rely on IL-10 and collaborate with Tregs to construct an immunosuppressive microenvironment. This collaborative interaction within the Treg–Breg axis creates a dual-layered mechanism for immune escape.

The highly coordinated immunosuppressive network in BCBM presents a rich array of therapeutic targets for clinical translation, yet also poses significant challenges. Clinical translation strategies can primarily focus on three levels, including reversing immunosuppression, reprogramming the tumor microenvironment, and developing synergistic combination therapies. Based on human single-cell data from BCBM and liver metastases, alternative immune checkpoints such as LAG3–galectin-3 are highly active in tumor and infiltrating cells, suggesting their potential as important complements to PD-1/PD-L1 inhibitors [130, 135]. Another major direction involves targeting immunosuppressive cells. The development of agents that deplete intratumoral Tregs or disrupt their function could fundamentally undermine a central pillar of immune suppression. Ultimately, the greatest translational potential lies in combination therapies. Given the highly cooperative nature of networks such as the Treg-Breg axis, any single-target intervention is likely to be counteracted by compensatory mechanisms. Therefore, designing combination strategies capable of simultaneously blocking multiple nodes is essential.

### Innate immune cells in the TME of BCBM

The brain microenvironment harbors distinct resident immune cell populations. These cells not only engage in intricate immune crosstalk with one another but also interact dynamically with cancer cells, creating a complex and highly coordinated immunological landscape (Fig. 4). Brain-associated macrophages play a dual and highly context-dependent role in BCBM. Brain-associated macrophages are primarily composed of two subgroups: microglia, which are tissue-resident macrophages of CNS origin, and border-associated macrophages (BAMs), including meningeal, perivascular, and choroid plexus macrophages [136]. This section delves into the specific role of BAMs in the microenvironment of BCBM. These macrophages facilitate the metastatic process of breast cancer cells to the brain by promoting angiogenesis, aiding cancer cell extravasation, and supplying the essential nutrients required for cancer cell survival. Research demonstrated that LYVE1^+^ perivascular macrophages in the lung play a crucial role in maintaining vascular tone through their interactions with vascular smooth muscle cells, which facilitate cancer cell metastasis via the bloodstream [137]. Moreover, BAMs are capable of secreting various factors, including vascular cathepsin-S, VEGF, and MMP9 [138, 139]. These secretions can increase the permeability of the BBB, thereby facilitating the entry of circulating tumor cells into the brain. The secretion of plasminogen activator inhibitory serpins (PAIS) by BAM can shield cancer cells from death signals and facilitate vascular co-option, thereby promoting the development of brain metastasis [140]. Upon breaching the BBB and infiltrating the meninges, cancer cells can co-localize with perivascular meningeal macrophages. Macrophages were induced to express the pro-survival neurotrophic factor, glia-derived neurotrophic factor (GDNF), which supports the survival of cancer cells in the nutrient-scarce environment of the leptomeninges [141].

In BCBM, tumor-associated neutrophils (TANs) display remarkable plasticity and dynamically modulate the brain microenvironment through both pro-metastatic and anti-tumor activities. Neutrophils are recruited to the brain prior to tumor cell arrival via tumor-derived factors, such as G-CSF, CXCL2 and TGF-β [142–144]. In turn, TANs release proteases, such as MMP-9 and lipocalin-2 (LCN2) to degrade the extracellular matrix (ECM) and disrupt the BBB, thereby enabling tumor cell extravasation [145]. During this process, TANs also promote tumor colonization by forming “neutrophil extracellular traps”, which capture circulating tumor cells, enhance their adherence to brain vessels, and activate pro-survival pathways in tumor cells [146–148]. In the brain microenvironment, stimulating factors such as IL-17, G-CSF and TGF-β drive TANs toward the “N2” pro-tumor phenotype [144, 149]. These N2 neutrophils secrete immunosuppressive factors and inhibit the activity of cytotoxic CD8^+^ T cells and natural killer (NK) cells through the expression of PD-L1 [150, 151]. However, studies have shown that treatment with IFN-β or TGF-β blockade can polarize neutrophils to an “N1” antitumor phenotype, characterized by enhanced tumor cell phagocytosis and recruitment of T cells via CXCL10 [151, 152].

As a core effector cells of innate immunity, NK cells are capable of directly killing tumor cells by recognizing stress ligands on the tumor cell surface. In breast cancer, NK cells can rapidly eliminate circulating tumor cells through the release of perforin and granzyme B, thereby inhibiting their survival in peripheral blood and brain tissue [153, 154]. However, the functionality of NK cells is significantly compromised by hypoxia, adenosine accumulation, and other environmental factors within the brain metastasis microenvironment. For instance, HERBC with brain metastases often form dense, spheroid tumor structures that impede the infiltration of NK cells [10]. In the CNS, N-acetylaspartate is released in response to brain inflammation. N-acetyltransferase 8-like (NAT8L) inhibits the cytotoxicity of NK cells and CD8^+^ T cells via N-acetylaspartate (NAA), thereby reducing brain inflammation while simultaneously undermining anti-tumor immunity [155]. Recent studies have explored and developed CAR-NK cell immunotherapy targeting HLA-G, which restores the natural killing function of NK cells by disrupting the interaction between HLA-G and NK cell inhibitory receptors [156, 157]. This strategy has effectively reversed the immunosuppressive state of NK cells in HER2BC with brain metastases and significantly inhibited tumor progression.

Myeloid-derived suppressor cells (MDSCs) are generated in the bone marrow of tumor-bearing hosts. The breast cancer TME promotes the recruitment and functional enhancement of MDSCs through chemokine signaling and metabolic adaptation. In breast cancer, cancer cells induce the recruitment and accumulation of MDSCs at the tumor site by secreting factors such as S100A9, CCL5, and so on [158, 159]. Cytokines such as CCL20 can induce the differentiation of granulocyte-monocyte progenitor cells via ligand-receptor interactions, thereby promoting the expansion of MDSCs [160]. In PIK3CA-mutated luminal breast cancer, cancer cells recruit MDSCs through the 5-lipoxygenase-dependent arachidonic acid metabolic pathway, and exclude cytotoxic T cells as well [161]. Cancer-associated fibroblasts (CAFs) also contribute by secreting chemokines like SDF1α, complement C3a, and C5a, CXCL1 and CXCL8, which facilitate the migration of MDSCs to tumor sites [162–164]. In BCBM, recruited MDSCs contribute significantly to both metastatic progression and immunosuppression. On one hand, MDSCs facilitate the invasion of tumor cells and their penetration through the BBB. In TNBC, MDSCs secrete cytokines like TGF-β and CXCL2, which enhances the stemness properties of breast cancer cells, thereby augmenting their metastatic potential [160, 165]. On the other hand, MDSCs actively participate in and support the formation of the pre-metastatic niche [166]. Moreover, MDSCs interact with the cellular components within the TME to suppress immune responses mediated by T cells and NK cells [167]. For instance, CD11b^+^Gr-1^+^ MDSCs, which are significantly expanded in BCBM, establish cell-to-cell contact with NK cells. Through this interaction, they inhibit the activation of NK cells by suppressing JAK3-mediated Stat5 activation [168].

The mechanisms underlying cellular crosstalk within the immunosuppressive microenvironment of BCBM are highly complex. Therapeutic strategies for clinical translation should focus on reprogramming the entire metastatic immune microenvironment rather than solely on killing tumor cells. On one hand, targeting key accomplice cells such as BAMs and TANs is critical. Cathepsin S or MMP9 inhibitors can be employed to reinforce the BBB, and when combined with anti-VEGF therapy, may suppress pro-angiogenic activity, thereby blocking early steps of tumor cell invasion and colonization. For highly plastic TANs, reprogramming from pro-tumoral N2 to anti-tumoral N1 phenotypes can be achieved using IFN-β in combination with TGF-β blockade. Concurrent administration of DNase I to degrade NETs may disrupt their role in facilitating tumor cell adhesion and survival. On the other hand, active measures are needed to reverse the functional suppression of effector immune cells. Small molecule inhibitors targeting the NAT8L/NAA pathway or adenosine A2A receptor antagonists can help neutralize immunosuppressive signals in the brain microenvironment. A more promising approach involves adoptive cell therapies, such as the infusion of HLA-G-targeting CAR-NK cells. Meanwhile, neutralization of MDSC recruitment and function, through S100A9 inhibition or combination with ARG1 inhibitors, may help alleviate immune suppression and create a permissive environment for the recovery of T and NK cell activity. Finally, combination therapeutic strategies should remain a central translational direction. By simultaneously targeting the pro-tumoral functions of specific cell subsets (e.g., BAMs, TANs, MDSCs), alleviating their suppression of effector cells (NK and T cells), and synergizing with immune checkpoint inhibitors, a multi-target and temporally-sequenced combinatorial approach can be constructed. Such a strategy is expected to significantly enhance treatment efficacy against BCBM.

### Specific cellular components in the TME of BCBM

**Fig. 5:**
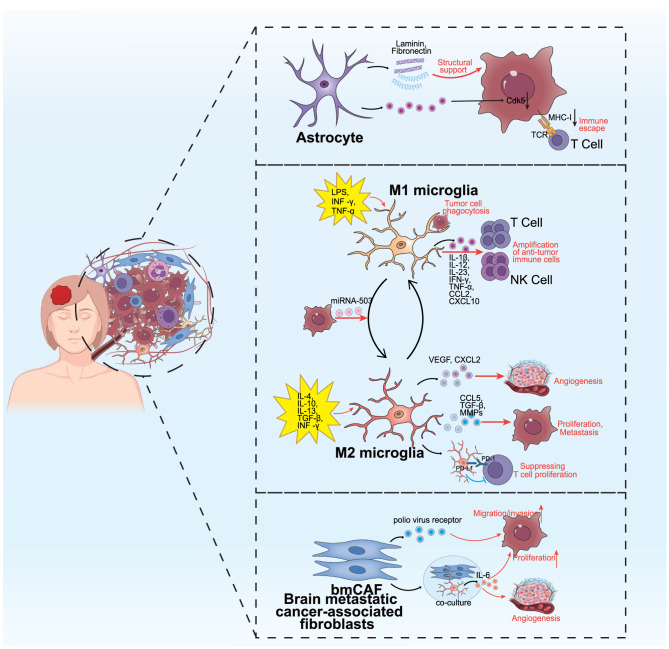
Unique cellular components and intercellular crosstalk in the tumor microenvironment of breast cancer brain metastases. The tumor microenvironment of brain metastases represents a sophisticated and intricate ecosystem. Brain metastases are not only populated by tumor cells, innate and adaptive immune cells, stromal cells, and the extracellular matrix but also incorporate some cellular components that are unique to the brain. These include astrocytes, microglia, and brain tumor-associated fibroblasts, collectively forming a distinctive intracranial ecological niche. Through diverse signaling molecules, tumor cells and various cellular components engage in intricate crosstalk, thereby establishing a singular tumor microenvironment specific to breast cancer brain metastases

The dynamic equilibrium between immune surveillance and suppression profoundly influences the progression of BCBM. This heterogeneity of the TME also stems from the intricate interplay between tumor cells and the specific cells of the CNS. As the “indigenous residents” of brain parenchyma, astrocytes and microglia are not only involved in BBB remodeling and metabolic support but also shape a unique immune ecology through cross-talk with tumor cells and immune components. Additionally, breast cancer cells that infiltrate the brain drive matrix remodeling and establish “outposts” for distal metastasis by recruiting CAFs. These specialized cellular components are interwoven with the immune network to form a multi-tiered regulatory axis, which collectively determines the colonization fate of BCBM. Figure 5 illustrates the distinct cellular components within the TME of BCBM, along with their intricate interactions.

#### Astrocyte

Within the network of glial cells, the role of astrocytes extends far beyond their traditional function as structural supporters. They actively participate in the remodeling of the brain metastasis microenvironment through both physical contact and the secretion of bioactive factors. On the one hand, astrocytes contribute to the construction of physical barriers, providing structural scaffolding for tumor cells while simultaneously offering essential nutritional support. For example, brain metastases in TNBC frequently form perivascular sheaths, whereas those in HER2BC tend to develop dense spheroids driven by autocrine tenascin-C production. Both of these metastatic patterns involve extensive engagement of astrocytes [10]. Astrocytes secrete laminin and fibronectin, which offer structural support to tumor cells, facilitating their survival and growth within the brain microenvironment. Meanwhile, astrocytes engage in bidirectional communication with cancer cells and drive their aggressive growth and metastasis by secreting a variety of cytokines, such as IFNα, TNF-α, CXCL12, TGF-β2, IGFBP2 and CHI3L1 [169–171]. Moreover, astrocytes exert immunomodulatory functions that aid breast cancer cells in evading immune surveillance. In brain metastases originating from breast cancer, astrocytes induce tumor cells to overexpress the neuron-specific cyclin-dependent kinase 5 (Cdk5). This overexpression subsequently inhibits the expression of MHC-I on the tumor cell membrane via the Irf2bp1-Stat1-importin α-Nlrc5 pathway, thereby preventing cancer cells from being recognized by T cells [172].

#### Microglia

As the resident macrophages of the brain, microglia play a complex and paradoxical role in BCBM, extending beyond a simplistic pro- or anti-tumor function. The classical M1 (anti-tumor)/M2 (pro-tumor) polarization model provides a preliminary framework for understanding their functional diversity. Theoretically, M1-polarized microglia may suppress tumor growth by releasing pro-inflammatory cytokines, phagocytosing tumor cells, and presenting antigens. In contrast, M2-polarized microglia are thought to support tumor progression through the secretion of growth factors, promotion of angiogenesis, and release of immunosuppressive mediators [173–175].

The M1 microglia is typically triggered and activated by pro-inflammatory mediators, such as lipopolysaccharide (LPS), interferon-γ (INF-γ) and tumor necrosis factor-α (TNF-α) [176, 177]. It secretes a plethora of pro-inflammatory cytokines and chemokines, including IL-1β, IL-12, IL-23, IFN-γ, TNF-α, CCL2, and CXCL10, which are pivotal in amplifying the infiltration of anti-tumor immune cells and in the elimination of cancer cells. In lung cancer cell brain metastasis events, M1 microglia have been observed to be activated by lipopolysaccharide and are capable of inducing apoptosis in metastatic lung cancer cells in vitro, demonstrating a dose- and time-dependent effect [178]. As a unique subset of macrophages, microglia primarily eliminate tumor cells through phagocytosis. Wu et al. discovered that tamoxifen can impede brain metastases by enhancing the M1 polarization of microglia and bolstering their anti-tumor phagocytic capacity [179]. Furthermore, M1 microglia also engage in adaptive immune responses by functioning as antigen-presenting cells. Katrina’s study, through single-cell RNA sequencing technology, jointly constructed genetic and humanized mouse models, found that microglia promote anti-tumor immune microenvironment in BCBM by supporting NK, NKT and T cell responses to BCBM [180]. The mutual activation of microglia-T cells is crucial for tumor inhibition.

In contrast, M2-type microglia are activated by anti-inflammatory cytokines, such as IL-4, IL-10, IL-13, and TGF-β, and secrete anti-inflammatory and immunosuppressive factors such as CCL5, TGF-β and MMPs, which facilitate tumor cell proliferation and the establishment of brain metastases. M2 microglia also play a role in the modulation of angiogenesis through the secretion of VEGF and CXCL2. Research has demonstrated a positive correlation between the polarization of M2 microglia and microvascular density in patients with glioblastoma [181, 182]. In gliomas, the inhibition of the CXCL2–CXCR2 signaling pathway has been shown to significantly diminish tumor volume. Furthermore, M2 microglia have been demonstrated to contribute to the establishment of a tumor immunosuppressive environment, which in turn facilitates tumor progression. A recent study has revealed that the absence of XIST in BC cells leads to an increase in the secretion of exosomal miRNA-503, which triggers M1–M2 polarization in microglia. This shift towards the M2 phenotype upregulates the expression of PD-L1 in microglia, consequently suppressing T cell proliferation and fostering the advancement of brain metastasis [183]. Metastatic tumor cells can facilitate their own growth and colonization by inducing the polarization of microglia from the M1 to the M2 phenotype.

However, overreliance on the M1/M2 paradigm obscures the true complexity of microglial functional states. Multiple single-cell and spatial omics studies have revealed that microglia do not adopt two discrete polarization states but instead dynamically transition along a continuous functional spectrum, often co-expressing both M1 and M2 markers. As a result, their net biological effect, whether promoting or suppressing tumor growth, is highly context-dependent, influenced by factors such as tumor subtype, metastatic stage, and crosstalk with other components of the tumor microenvironment. For instance, on the pro-metastatic side, breast cancer cells can reprogram microglia via exosomal miR-503, driving a shift toward an M1–M2 hybrid phenotype. This transition involves upregulation of PD-L1 in microglia through STAT3/NF-κB signaling, leading to T cell suppression and facilitated brain metastasis. This axis has been consistently recapitulated and reinforced in recent mechanistic studies and reviews. Conversely, there is also evidence suggesting that microglia can enhance anti-tumor immunity and restrict the growth of BCBM. These seemingly contradictory findings collectively underscore the need to move beyond the M1/M2 dichotomy. Future efforts should leverage single-cell and spatial multi-omics technologies to precisely map the continuous functional states of microglia across different BCBM subtypes and treatment contexts, and to identify the key molecular hubs that govern their switch between pro- and anti-tumor roles. Only through such nuanced understanding can microglia-targeted therapies transition from conceptual promise to clinically viable and precise interventions.

#### Brain metastatic cancer-associated fibroblasts (bmCAFs)

bmCAFs play a pivotal role in the progression of BCBM as well. Existing research has demonstrated that bmCAFs can promote the formation of large metastatic lesions through multiple mechanisms, including the secretion of signaling factors, involvement in shaping distinct tumor structures, and metabolic reprogramming.

Distinct from fibroblasts found in primary tumors, bmCAFs can be stimulated to secrete polio virus receptor under hypoxic conditions, thereby enhancing the migration and invasion of TNBC cells. This enhancement is achieved by altering intercellular connectivity and modulating the dynamics of the actin cytoskeleton [184]. Breast cancer cells can acquire enhanced migratory capacity, thereby more readily penetrating the BBB. Moreover, a co-culture model of bmCAFs and astrocytes revealed that the astrocytes were induced to produce pro-inflammatory factors such as IL-6, which amplified pro-angiogenic signals and promoted tumor cell proliferation and angiogenesis.

Different subtypes of breast cancer have distinct tumor architecture and TME traits, which in turn endow brain metastases with unique colonization patterns and spatial characteristics. By leveraging single-cell transcriptomic analyses of the TME, alongside mouse models and patient tissue samples, researchers have uncovered that TNBC and HER2BC cancers display divergent tumor structures and are shaped by different tumor-ECM interactions and autocrine growth mechanisms [10]. TNBC usually develops a perivascular sheath that maintains extensive contact with astrocytes and microglia. This perivascular colony structure facilitates superior access to enough oxygen, adequate nutrients, and basement membrane anchorage, thereby promoting survival and growth. In contrast, HER2BC usually forms dense spherical structures, which pushes stromal cells to the periphery. This pattern more closely resembles the archetypal growth observed in primary tumors. These distinct structural profiles elicit varying responses from Alzheimer’s disease-associated microglia, with the GAS6-AXL signaling pathway implicated and exerting divergent effects. In TNBC, the GAS6-AXL signaling within cancer cells seem to foster cell survival, whereas in HER2BC, elevated GAS6 expression triggers pro-tumor AXL signaling.

Collectively, both astrocytes and bmCAFs significantly contribute to the formation of the brain metastatic microenvironment, albeit exhibiting distinct tumor architectures and glial responses across different molecular subtypes. Targeting specialized cellular components, including astrocytes, bmCAFs, and microglia, requires a coordinated approach based on “cell type–structural niche–therapy” matching. Adopting a “reprogramming rather than eliminating” strategy, combined with evaluating therapeutic combinations, including ADCs, immune checkpoint inhibitors (ICIs), and metabolic inhibitors, within breast cancer subtype-specific contexts, holds promise for developing effective treatments.

## Current diagnostic and therapeutic landscape and future horizons

### Molecular markers for screening, early diagnosis and risk prediction of BCBM

The interval during which cancer cells disseminate from the primary tumor and migrate to distant sites, particularly the brain, to form large metastases represents a crucial time window for effective clinical intervention and is pivotal for preventing brain metastasis. During this period, the identification and application of effective molecular biological markers hold significant value for the early detection of metastasis. Liquid biopsy, as an important diagnostic modality, can effectively complement the limitations of traditional detection methods. The presence of cancer cells and their associated biomolecules in the bloodstream, if effectively detected or quantitatively analyzed, can facilitate early diagnosis of brain metastases.

Circulating tumor cells (CTCs) hold promise as potential blood-based biomarkers for the diagnosis and management of BCBM. CTC counting has been demonstrated to predict the metastatic potential in breast cancer. For instance, Giuliano et al. investigated CTC counts in 492 patients with advanced breast cancer and analyzed their correlation with metastatic characteristics. The results revealed that, compared to patients with low CTC counts, those with elevated CTC levels (≥5 CTCs) exhibited a significantly higher number of metastatic sites and developed new metastatic lesions at a much faster rate [185]. Besides, the longitudinal study results indicate that continuous long-term monitoring of the dynamic changes in CTCs is superior to a single CTC count, which provides more comprehensive and valuable information for predicting disease progression [186]. Ring et al. conducted RNA-Seq on fresh metastatic tumor biopsies, CTCs, and peripheral blood samples from 19 newly diagnosed patients with metastatic breast cancer [187]. Their study demonstrated that RNA-Seq of CTCs can serve as an alternative biomarker for large breast cancer metastasis, providing clinically relevant insights into disease biology and potential therapeutic targets. This approach not only enhances the understanding of metastatic mechanisms but also offers a minimally invasive means to monitor disease progression and response to treatment. However, it is noteworthy that due to the limited specificity of CTCs for brain metastases, they are more suitable as a dynamic monitoring tool rather than a standalone diagnostic indicator.

Cell-free or circulating tumor DNA (ctDNA) in the blood of cancer patients was first described in 1948 and has since emerged as a potential biomarker for metastasis. A recent study has demonstrated that ctDNA from metastatic brain tumors, including mutations in ALK and MDM2, can be used to effectively differentiate metastatic brain tumors from gliomas [188]. The detection of ctDNA in CSF samples achieves an accuracy of over 90% [189]. However, its widespread application remains limited due to the invasive nature of sample collection. In contrast, methylation markers derived from neuronal or glial cells in plasma cell-free DNA (cfDNA) show potential for early localization of brain metastases and offer a safer sampling alternative, though their clinical utility still requires validation through multicenter prospective studies [190].

The development of brain metastases in breast cancer is associated with specific proteins and metabolites. Carcinoembryonic antigen (CEA) and carbohydrate antigen 153 (CA153) are the most commonly used blood markers for breast cancer management. Within the BCBM microenvironment, periostin (POSTN) is produced by POSTN-positive CAFs and promotes cancer cell proliferation by positively regulating the HGF-MET signaling pathway [191]. Studies have demonstrated that monitoring POSTN for predicting distant metastasis in breast cancer performs comparably to CA153, suggesting that POSTN can serve as a novel biomarker to complement CEA and CA153 in predicting BCBM [192]. Similarly, bioinformatic analyses have identified several proteins, including CD44, COL1A2, MMP14, POSTN, and SOX9, as potential diagnostic biomarkers for patients with BCBM [193].

In addition, in breast cancer, hundreds of millions of extracellular vesicles selectively alter the levels of their contents, which include DNA, RNA, microRNA, long-chain non-coding RNAs (lncRNA), circular RNAs, lipids, proteins, and various metabolites. These biomolecules play a pivotal role in the metastatic process [194]. As exposed to patient body fluids, these secreted biomolecules hold great potential to serve as non-invasive biomarkers for BCBM. Biofluid metabolomics is anticipated to play a significant role in the early diagnosis of BCBM. A recent animal model study investigating BCBM revealed that these metastases possess a distinct metabolite fingerprint, which can potentially be identified through urine metabolomics [195]. In a population cohort comprising 88 patients with breast cancer metastasis, serum metabolomics analysis uncovered substantial alterations in the expression levels of numerous metabolites [196]. Specifically, the concentrations of 11 metabolites, including alanine, sphingosine, fructose, fumaric acid, glycine, lactic acid, phenylalanine, pyroglutamic acid, serine, threonine, and valine, were significantly elevated in patients with breast cancer metastases compared to the control group. Moreover, the identification models of 5 and 10 metabolites demonstrated remarkable predictive accuracy for BCBM, achieving rates of 94.6% and 95.2%, respectively. The field of proteomics and metabolomics has witnessed exponential growth over the past decade. The detection and quantification of differentially-expressed products associated with BCBM diagnosis hold great promise for addressing the challenge of identifying diagnostic markers for BCBM. In Table 3, we list potential molecular markers that show promise for early screening, diagnosis and risk prediction of BCBM.

**Table 3:** Potential diagnostic biomarkers for breast cancer brain metastasis

Biomarkers	Sample	Main Use	Evidence	Diagnostic Performance	Stage of validation	Translational readiness	Reference
Circulating tumor cells (CTC)	Peripheral blood	Prognosis or therapy monitoring	Elevated CTC levels predict higher number of metastatic sites and faster new lesion development.	Threshold ≥5 CTCs associated with shorter PFS; dynamic monitoring more predictive than single measurement.	Retrospective clinical cohort	Early clinical application	[185, 186, 197]
Circulating tumor DNA (ctDNA)	Cerebrospinal fluid	Diagnosis or monitoring of breast cancer leptomeningeal metastasis	Detectable in CSF of breast cancer leptomeningeal metastasis patients; rarely detected in non-brain metastases cases	Sensitivity 93%, specificity 100%, accuracy 94%.	Small-scale clinical studies (*n* = 30)	Promising precision diagnostic tool; requires large-scale validation	[189, 198]
Matrix metalloproteinase (MMP)-9	Serum	Risk association	Serum MMP-9 level was significantly elevated in BCBM patients (*p* = 0.0062), which could accurately differentiate patients with brain metastases.	Significant difference (*p* = 0.006), but limited sensitivity or specificity data.	Clinical cohorts	Low (exploratory)	[199]
Neuron-specific enolase (NSE)	Serum	Suggestive indicator of possible brain metastasis	Serum NSE levels were significantly elevated in BCBM patients (*p* = 0.0051), which could serve as an independent prognostic factor in BCBM patients	p = 0.005; independent prognostic factor.	Clinical cohorts	Low (exploratory)	[199]
Thrombospondin-1 (THBS1)	Plasma/tissue	Risk prediction	Plasma THBS1 elevated in HER2-enriched patients at higher brain metastases risk.	High-plasma THBS1 levels were specifically associated with an increased occurrence of brain metastasis in HER2-enriched patients (*p* = 0.041).	Clinical cohorts	Low–moderate (needs external validation)	[200]
Periostin (POSTN)	Serum	Prediction of distant metastasis	Secreted by CAFs; elevated levels associated with distant metastasis.	AUC≈0.78, comparable to CA153.	Clinical cohorts	Moderate (validated but not routine)	[191, 192]
Dehydrogenase (LDH), LDH-A	Serum	Risk prediction	In TNBC, LDH-A expression and baseline LDH slope correlate with brain metastasis occurrence and brain metastasis free survival (BM-FS).	Correlation significant.	Clinical cohorts	Moderate	[201]
GATA3 and SOX-10	Tissue (brain metastasis biopsy)	Prediction of prognosis	≥1 positive in ~100% in TNBC metastases, single marker 70–95%	The sensitivity and specificity of Sox10 and GATA-3 in metastatic TNBC were 68.2% and 32.6%, 86.4% and 41.3%, respectively.	Clinical cohorts	High (routine pathology use)	[202, 203]
Retinoic acid receptor responder 2 (RARRES2)	Tissue	Risk prediction	RARRES2 is down-regulated in TNBC and mediates the development of brain metastases by regulating lipid metabolic reprogramming.	Clinical diagnostic metrics not reported.	Preclinical	Low (exploratory)	[204]
miR-4428	Plasma	Discrimination of BCBM	Plasma miR-4428 discriminates BCBM vs non-BCBM	AUC = 0.779.	Retrospective clinical cohort	Moderate	[194]
miR-4480	Plasma	Discrimination of BCBM	Plasma miR-4480 distinguishes BCBM patients, achieving an AUC of 0.781.	AUC = 0.781.	Retrospective clinical cohort	Moderate	[194]
lncRNA GS1-600G8.5	Exosomes	Prediction of BBB disruption.	The lncRNA GS1-600G8.5 is highly expressed in highly metastatic brain cells and their exosomes, and it promotes breast cancer brain metastasis by disrupting the blood–brain barrier system.	Preclinical and mechanistic studies, diagnostic accuracy not reported.	Preclinical	Low (exploratory)	[108]
circBCBM1	Tissue/plasma	Prediction of BM-FS; discrimination of BCBM	Promotes BCBM; high expression linked with shorter BM-FS	Preclinical and mechanistic studies, diagnostic accuracy not reported.	Preclinical	Low (exploratory)	[118]
circKIF4A	Tissue	Risk prediction	circKIF4A/miR-637/STAT3 axis promotes BM; silencing reduces BM ability.	Mechanistic and patient sample studies, diagnostic accuracy not reported.	Preclinical	Low (exploratory)	[205]
Metabolites	Serum/plasma	Early detection ofo BCBM	The model comprising 15 metabolites achieved an accuracy of 96.9% in predicting BCBM.	Exploratory model accuracy ≈96.9%	Clinical cohorts	Moderate (requires external validation)	[206]

Although liquid biopsy and multi-omics biomarkers show considerable potential for the early prediction of BCBM, the clinical translation still faces multiple challenges. On one hand, the BBB reduces the sensitivity of detection methods relying on plasma or exosomes. Although CSF-based assays offer higher specificity, their invasive sampling procedure limits routine clinical use. Moving forward, optimizing sampling strategies and combining plasma- and CSF-derived biomarkers may improve both sensitivity and specificity. Secondly, the lack of consistency in sample processing, detection platforms, and cut-off values across different studies hinders the comparability of results. It is essential to promote standardization of detection technologies and operational protocols to ensure cross-platform reproducibility. Integrating multi-dimensional data, such as ctDNA, CTCs, exosomes, and metabolomic profiles, to develop multi-omics predictive models, coupled with artificial intelligence (AI)- assisted interpretation, could also facilitate early detection of BCBM. Finally, large-scale, multi-center prospective clinical trials are needed to clarify the practical utility of these biomarkers in early screening, risk stratification, and therapeutic decision-making. Only through dual advances in both technological innovation and clinical application can liquid biopsy and multi-omics biomarkers truly become feasible tools for the early diagnosis and precise prevention of BCBM.

### Current therapeutic landscape of BCBM

Currently, the standard first-line treatment regimens for breast cancer are primarily driven according to the molecular subtypes. Patients with the HER2BC routinely receive anti-HER2 monoclonal or dual antibodies combined with chemotherapy; those with the HR+/HER2- subtype are treated upfront with CDK4/6i plus endocrine therapy; while patients with TNBC typically receive chemotherapy with or without immunotherapy. However, the development of brain metastases presents unique therapeutic challenges. The BBB significantly restricts the penetration of multiple conventional systemic agents into the brain. Local interventions such as surgery and radiotherapy are often ineffective in addressing occult micrometastases and are associated with high recurrence rates, making them inadequate substitutes for systemic therapy. Therefore, there is an urgent need to overcome the limitations imposed by the BBB, extend intracranial disease control, and develop innovative therapeutic agents or treatment strategies.

In recent years, a variety of novel agents and therapeutic strategies have emerged and gradually demonstrated potential in treating BCBM.

HER2BC constitutes approximately 20% to 30% of all breast cancer cases, and around 30% of these may develop brain metastases. HER2-targeted agents such as trastuzumab and pertuzumab exert the therapeutic effects by inhibiting receptor signaling and enhancing immune-mediated cytotoxicity. A retrospective study by Park et al. indicated that patients treated with trastuzumab had a significantly longer median time to the development of BCBM compared to those who did not receive the drug (15 months vs 10 months, *p* = 0.035), as well as a significantly prolonged median OS (14.9 months vs 4.0 months, *p* = 0.0005) [207]. Similar conclusions were reported in a study by Dawood et al. [208]. For patients with active BCBM that progressed after radiotherapy, the result of PATRICIA trial demonstrated that high-dose trastuzumab in combination with pertuzumab received a CNS objective response rate (ORR) of 11%, with a median OS of 27.2 months [209]. However, most of the current evidence is derived from retrospective studies or single-arm trials, which lack the support of randomized controlled trials (RCTs) data and are susceptible to biases. Moreover, combination therapy with monoclonal antibodies often leads to disease stabilization or delayed progression rather than tumor regression, indicating that ORR remain limited. Additionally, the efficacy of these agents largely depends on the disruption of BBB in metastatic lesions, which may restrict their activity in patients with intact BBB.

HER2-targeted tyrosine kinase inhibitors (TKIs), owing to their low molecular weight and high lipophilicity, can more effectively cross the BBB. These agents have demonstrated promising efficacy in RCTs, significantly improving CNS progression-free survival (PFS) and OS. However, the majority of clinical data regarding TKI efficacy currently originate from single-arm or early-phase trials [210]. Furthermore, the emergence of secondary mutations may lead to the development of drug resistance. Additionally, there is still a lack of predictive biomarkers capable of identifying patient populations most likely to benefit, which hampers the advancement of individualized treatment strategies [211].

CDK4/6i serve as the first-line therapeutic agents for patients with HR^+^/HER2^−^ breast cancer, exerting their therapeutic effects through cell cycle arrest, immune and metabolic regulation. Growing insights into the TME of BCBM suggest that CDK4/6 inhibitors may contribute to the reprogramming of the immune microenvironment. As previously mentioned, BCBM is characterized by CD8^+^ T cell exhaustion and inadequate infiltration. CDK4/6 inhibitors can enhance MHC-I expression, thereby indirectly improving CD8^+^ T cell recognition [212]. Animal models have demonstrated that treatment with abemaciclib restores effector function in CD8^+^ T cells and alleviates their exhausted state. Additionally, Tregs exhibit high sensitivity to CDK4/6 inhibition, which suppresses Treg proliferation [213]. In patients with BCBM characterized by CD8^+^ T cell exhaustion or Treg dominance, CDK4/6i may effectively mitigate the immunosuppressive state, thereby establishing a more favorable foundation for immunotherapy [208].

Current evidence supporting the efficacy of ICIs in BCBM remains largely derived from small-sample or single-arm clinical trials. A phase II trial evaluating the combination of SHR-1316, bevacizumab, and cisplatin/carboplatin in TNBC patients with active brain metastases demonstrated promising outcomes, with a CNS ORR of 65.2%, alongside improvements in PFS and OS [214]. Bispecific antibodies (BsAbs) and chimeric antigen receptor-T (CAR-T) cell therapy represent emerging areas in immunotherapy, and their application in BCBM is still in the early exploratory stage. BsAbs simultaneously bind to antigens on the surface of tumor cells and activating receptors on the surface of immune cells via a single molecular structure, “anchoring” immune effector cells near tumor cells to specifically trigger localized immune-mediated killing. Currently, HER2-targeted BsAbs (e.g., zanidatamab) are undergoing clinical trials and have initially demonstrated efficacy against BCBM [215–218]. However, similar to monoclonal antibodies, BsAbs are typically large-molecular-weight proteins that cannot readily penetrate the intact BBB passively. Additionally, tumor cells may evade BsAb recognition by downregulating the expression of target antigens, which can lead to suboptimal therapeutic efficacy or short-lived treatment responses. CAR-T therapy involves genetically engineering a patient’s autologous T cells to express chimeric antigen receptors that recognize tumor-specific antigens, enabling efficient tumor cell killing [219, 220]. Currently, the standard administration route for CAR-T therapy is intravenous infusion. A critical challenge, however, lies in the number of CAR-T cells that can successfully home to brain metastatic lesions. Meanwhile, in the solid tumor microenvironment, CAR-T cells are prone to exhaustion, with their proliferative capacity and cytotoxic function diminishing over time and the immunosuppressive TME is a major underlying barrier. Beyond these issues, the potential neurotoxicity of these therapies cannot be overlooked. One of the primary toxicities associated with both BsAbs and CAR-T therapy is cytokine release syndrome (CRS) and immune effector cell-associated neurotoxicity syndrome (ICANS) [221, 222]. Furthermore, effective tumor killing may trigger significant local inflammatory responses and cerebral edema. These lead to increased intracranial pressure, complications that require proactive medical management. Therefore, future efforts should focus on optimizing local delivery routes, such as intracerebral CAR-T administration or combination with focused ultrasound to open the BBB, to enhance intracranial drug concentrations. Additionally, combination strategies, such as ICIs, may help counteract the immunosuppressive microenvironment and antigen escape. Multitargeting approaches, along with toxicity early-warning systems and graded management protocols, could further mitigate neuroinflammatory risks. These integrated strategies hold promise as potential solutions.

PARP inhibitors are targeted therapeutic agents designed to impair DNA damage repair, and are primarily used in breast cancer patients with BRCA1/2 mutations. A case report documented a patient with TNBC and metastases to the lungs and brain who derived long-term benefit from olaparib monotherapy [223]. Although dedicated clinical data on the efficacy of PARP inhibitors in BCBM remain limited, systematic reviews and meta-analyses suggest that PARP inhibitors significantly improve ORR (relative risk≈2.00, *p* = 0.02) and PFS (hazard ratio≈0.68, *p* < 0.0001) compared to chemotherapy in metastatic TNBC [224]. These analyses included a subset of patients with BCBM. Given that TNBC is a subtype with a high risk of brain metastases, these findings provide a sound biological and clinical rationale for further exploration of PARP inhibitors in BCBM patients. Future studies specifically evaluating the efficacy of PARP inhibitors in BCBM patients are warranted.

In recent years, ADCs and peptide–drug conjugates (PDCs) have emerged as innovative precision therapeutics and become a major focus of research for the treatment of BCBM, owing to their unique drug delivery mechanisms and potential to cross the BBB.

ADCs represent a class of targeted agents that combine the specificity of monoclonal antibodies with the potent cytotoxicity of payload compounds. Their structure consists of three key components: an antibody, a cytotoxic payload, and a linker [225, 226]. The antibody selectively binds to tumor-associated antigens such as HER2 or TROP2, enabling precise drug delivery. The ultra-potent cytotoxic payload typically consists of DNA topoisomerase I inhibitors (such as DXd, SN-38) or microtubule inhibitors, which are orders of magnitude more potent than conventional chemotherapy agents. The linker serves as a bridge and regulates the stability and release of the toxin.

Multiple clinical trials in recent years have demonstrated the remarkable efficacy of ADCs in treating BCBM. Agents such as DS-8201a and T-DXd (targeting HER2), as well as sacituzumab govitecan (targeting TROP-2), have shown substantial antitumor activity and a favorable safety profile, providing renewed therapeutic options for this patient population [23, 227–229].

Similarly, PDCs employ smaller molecular-weight peptides as targeting ligands, offering superior tissue penetration and reduced immunogenicity. [230] ANG1005 represents the first PDC specifically engineered for BCBM, featuring three paclitaxel molecules covalently conjugated to the Angiopep-2 peptide. This engineered peptide selectively targets lipoprotein receptor-related protein 1 (LRP1), a receptor overexpressed on both BBB endothelial cells and brain-metastatic breast cancer cells. Leveraging LRP1-mediated transcytosis, ANG1005 achieves active BBB traversal and precisely delivers paclitaxel to intracranial tumor microenvironments, enabling localized drug accumulation. Phase 2 clinical trial data revealed clinical benefit in 70% of treated patients, with objective intracranial responses including partial remission in 14% and sustained disease stabilization in 56% of responders [231]. In Table 4, we compare and summarize the differences between ADCs and PDCs.

**Table 4:** The differences between antibody-drug conjugate (ADC) and peptide-drug conjugate (PDC)

	ADC	PDC	Key differences/Implications
Structure	Monoclonal antibody (mAb) + linker + cytotoxic payload	Engineered peptide + linker + cytotoxic payload	PDC replaces the large mAb with a small synthetic peptide scaffold.
Targeting moiety	Monoclonal antibody	Functional peptide	Molecular weight,molecular properties.
Molecular weight	High	Low	PDCs demonstrate superior tissue penetration and faster clearance kinetics.
Penetrability	Moderate-low (limited in dense tumors/BBB transit)	High (enhanced diffusion capability)	PDCs exhibit enhanced BBB crossing potential.
Target affinity	High (nanomolar/sub-nanomolar)	Moderate (micromolar range, variable)	ADCs achieve superior target engagement accuracy; while PDCs may show higher off-target risks.
Drug loading	High (typically 2–8 molecules/antibody)	Low (typically 1–2 molecules/peptide; e.g., ANG1005 carries 3 paclitaxel units)	ADCs deliver higher cytotoxic payload per molecule.
Mechanism of action	1. The antibody binds to specific antigens on the surface of target cells. 2. The antibody-antigen complex is internalized into the cell through endocytosis. 3. The linker is cleaved in the lysosome (in an acid or enzyme-dependent manner), releasing the cytotoxic payload (toxin). 4. The released toxin exerts its cytotoxic effect to kill the target cell.	1. The peptide binds to specific receptors on the cell surface. 2. The peptide-receptor complex is either internalized into the cell via receptor-mediated endocytosis or transported across biological barriers (e.g., the blood-brain barrier, BBB) through transcytosis.3. The cytotoxic payload (toxin) is released inside the cell or within lysosomes.4. The released toxin exerts its cytotoxic effect to kill the target cell.	PDC has unique potentials in actively crossing BBB.
Representative targets	HER2, TROP2	LRP1	PDCs focus on receptors critical for barrier penetration & CNS targeting.
Drug examples	DS-8201a (HER2-ADC); T-DXd (HER2-ADC); Dato-DXd (Trop-2-ADC); SG (Trop-2-ADC)	ANG1005 (GRN1005)	ADCs are clinically validated; PDCs are in early-phase trials.
BCBM advantages	T-DXd: > 70% CNS ORR in HER2+ BCBM; SG: activity in TNBC BM	ANG1005: 70% clinical benefit rate; intracranial ORR (14% PR + 56% SD)	Both show promise in clinical efficacy.
Key strengths	High target specificity; potent payload delivery; robust clinical validation.	Deep barrier penetration; low immunogenicity; easier synthesis; lower cost potential	/
Limitations	Lower BBB penetration; linker instability risk (off-target toxicity); complex manufacturing	Lower target affinity may lead to off-target effects; lower drug loading capacity; peptides are prone to be degraded by proteases	/

Regarding current therapeutic strategies for BCBM, Table 5 summarizes key efficacy data from clinical trials involving monoclonal antibodies, TKIs, CDK4/6 inhibitors, immunotherapy, as well as ADCs and PDCs.

**Table 5:** Clinical trial studies on the latest treatment of breast cancer brain metastases

Trial/ID	Regimen	Modality	Phase	Primary outcome	Reference
NCT01325207	intrathecal trastuzumab	anti-HER2 mAb (intrathecal)	Phase 1/2 (single-arm), breast cancer with leptomeningeal metastases	median PFS = 2.8 months; median OS = 10.5 months	[232]
LANDSCAPE (NCT00967031)	Lapatinib + capecitabine	HER2 TKI	Phase 2 (single-arm)	objective CNS response rate = 65.9% (all were partial responses).	[233]
TBCRC022 cohort 3A	Neratinib+capecitabine (lapatinib-naïve)	HER2 TKI	Phase 2 (single-arm)	CNS ORR = 49%; median PFS = 5.5 months; median survival = 13.3 months	[234]
TBCRC022 cohort 3B	Neratinib+capecitabine (prior lapatinib)	HER2 TKI	Phase 2 (single-arm)	CNS ORR = 33%; median PFS = 3.1 months; median survival = 15.1 months	[234]
NALA Trial-CNS subgroup	Neratinib + capecitabine vs lapatinib + capecitabine	HER2 TKI	Phase 3 (randomized)	median PFS = 7.0 vs 5.4 months; median OS = 23.8 vs 18.7 months; ORR = 40.7% vs 32.1%	[235]
PERMEATE (NCT03691051)	Pyrotinib + capecitabine (RT-naïve BM or progression after RT)	HER2 TKI	Phase 2 (single-arm)	median PFS = 10.9 vs 5.7 months; median OS = 35.9 vs 30.6 months; median CNS-PFS = 13.6 vs 5.7 months	[236]
JPBO (NCT02308020)	Abemaciclib	CDK4/6 inhibitor	Phase 2 (single-arm)	intracranial clinical benefit rate (iCBR) = 24%	[237]
NCT02886585	Pembrolizumab	PD-1 inhibitor	Phase 2 (single-arm), breast cancer with leptomeningeal metastases	37% patients had intracranial benefit	[238]
DESTINY-Breast12 (NCT04739761)	T-DXd	HER2 ADC	Phase 3b/4 (single-arm)	12-month PFS = 61.6%; 12-month CNS-PFS = 58.9%; ORR = 49.7% (stable BMs) or 54.7% (active BMs)	[5]
TUXEDO-1 (NCT04752059)	T-DXd	HER2 ADC	Phase 2 (single-arm)	median PFS = 21 months	[228]
DEBBRAH (NCT04420598)	T-DXd	HER2 ADC	Phase 2 (single-arm)	median OS = 13.3 months; median PFS = 8.9 months; clinical benefit rate (CBR) = 71.4%	[239]
KAMILLA (NCT01702571)	T-DM1	HER2 ADC	Phase 3b (single-arm)	overall response rate = 21.4%; median PFS = 5.5 months	[240]
NCT05769010	SHR-A1811	HER2 ADC	Phase 2 (multi-arm)	Intracranial ORR = 84%	[241]
ASCENT (NCT02574455)	Sacituzumab govitecan (SG)	TROP-2 ADC	Phase 3 (randomized)	median PFS = 2.8 months; median OS = 7.0 months; ORR = 3%; CBR = 45%	[242]
NCT02048059	ANG1005 (GRN1005)	PDC	Phase 2 (single-arm)	patient benefit (stable disease or better) = 77% (intracranial) and 86% (extracranial); intracranial ORR = 15% (investigator)	[231]
NCT05293964	SIM0270	estrogen receptor degrader	Phase 1	ORR = 12.9%	[243]
HER2CLIMB (NCT02614794)	Trastuzumab+capecitabine+tucatinib	anti-HER2 mAb + HER2 TKI	Phase 3 (randomized)	PFS at 1 year = 24.9%; PFS = 7.6 months	[20]
HER2CLIMB-02 (NCT02614794)	Tucatinib+T-DM1 vs T-DM1	HER2 TKI+HER2 ADC	Phase 3 (randomized)	median PFS = 9.5 months; ORR = 42%	[244]

### Future innovative and translational strategies BCBM therapy

In the preceding sections, we demonstrated that processes such as EMT, ECM remodeling, angiogenesis, and metabolic reprogramming are critical steps in the occurrence of metastasis. These mechanisms not only elucidate how tumor cells detach from the primary site, breach the BBB, and colonize the brain, but also offer potential avenues for future therapeutic interventions. However, the true challenge lies in effectively translating these mechanistic insights into clinically actionable strategies for precision therapy.

In different molecular subtypes of breast cancer, EMT may be driven by distinct signaling pathways. For instance, HER2BC more frequently exhibits activation of the TGF-β/SMAD-SNAIL axis, whereas TNBC often demonstrates activation of the Wnt/β-catenin pathway. These differences suggest that future translational research should place greater emphasis on patient stratification and the development of companion diagnostic tools. AI technologies, integrated with multi-omics data, may facilitate the establishment of an EMT scoring system to identify patient populations most likely to benefit from specific treatments. However, directly targeting upstream EMT pathways is often associated with significant toxicity and low selectivity due to their broad physiological roles, resulting in limited efficacy of monotherapies and challenges in clinical application [245]. A more feasible alternative may involve the indirect modulation of downstream effectors or key transcription factors of EMT, for example, targeting core regulators such as Snail, Twist, and Zeb with drugs or inhibitors, or employing molecular tools like microRNAs and long non-coding RNAs to selectively intervene in the transcriptional network of EMT. In Table 1, we summarize various transcription factors, microRNAs, and pharmacological agents that have been proposed in current studies to modulate the EMT process. Furthermore, previous studies provide a theoretical foundation for the potential of combining EMT inhibitors with radiotherapy, anti-HER2 agents, or ADCs to overcome drug resistance and improve intracranial disease control [246, 247]. For example, acquired resistance to trastuzumab can induce EMT and transition toward a TNBC-like phenotype, indicating that inhibiting EMT and associated plasticity may restore sensitivity to anti-HER2 therapy [248]. Therefore, a mechanism-informed approach utilizing subtype-specific EMT-targeting agents in combination with other treatment modalities represents a promising strategic direction for the treatment of BCBM.

ECM remodeling promotes metastasis by altering matrix mechanics and structure through modifying enzymes such as MMPs and the LOX family. However, the clinical failure of first-generation broad-spectrum MMP inhibitors served as a cautionary tale for subsequent drug development [249, 250]. Systematic reviews and historical drug analyses revealed that marimastat, batimastat, prinomastat, among others, generally failed in Phase III trials for solid tumors, exhibiting limited efficacy and dose-limiting toxicities such as musculoskeletal pain syndrome. Key reasons included overly broad targeting, insufficient pharmacokinetic exposure, and excessive inhibition of MMP physiological functions. Similarly, the LOXL2 inhibitor simtuzumab did not demonstrate significant benefits across multiple oncology studies [251, 252]. Therefore, for ECM-targeted interventions to succeed clinically, a shift toward precise target and patient selection is imperative, moving away from a one-size-fits-all inhibitory approach. Prioritizing specific targets and implementing subtype stratification should become primary strategies in BCBM management. In high-risk subtypes with frequent brain metastases, such as HER2BC and TNBC, a systematic evaluation of the correlation between ECM-related molecular activity and intracranial outcomes is essential. By matching specific targets with specific patient populations and adopting inhibitors with single-enzyme selectivity or activity-specific profiles instead of broad-spectrum strategies, there is potential to improve clinical outcomes.

Angiogenesis inhibitors, like bevacizumab, can effectively reduce tumor angiogenesis by inhibiting the VEGF signaling pathway, thereby significantly improving the prognosis of patients with BCBM. A Phase 1 clinical trial demonstrated that the combination of trastuzumab, lapatinib, and bevacizumab achieved disease stabilization (SD) for at least six months and partial or complete response (PR/CR) in 50% of patients with BCBM [253].

The mechanisms by which breast cancer cells cross BBB or BCFB involve multiple pathways, including disruption of tight junctions, regulation of signaling pathways, immune molecule-mediated facilitation, and exosome-driven transcellular transport. These mechanistic insights also offer potential avenues for clinical intervention. On one hand, molecules associated with barrier disruption may serve as biomarkers for identifying high-risk patients with brain metastasis, enabling early risk stratification and dynamic monitoring. On the other hand, limiting barrier damage could help reduce metastatic colonization, while controlled and moderate modulation of barrier function may enhance the delivery of antitumor drugs into the central nervous system. For example, Table 2 lists several key molecules that regulate the permeability and integrity of the brain barrier. Targeted inhibition of these molecules may help reduce tumor cell entry into the brain and early colonization. An alternative clinically feasible strategy involves combining agents that inhibit BBB permeability with systemically administered drugs capable of crossing into the brain, thereby simultaneously promoting barrier homeostasis and tumor suppression. Physical approaches capable of instantaneous and reversible opening of the BBB offer an engineering solution to the delivery challenge. Magnetic resonance-guided focused ultrasound (MRgFUS) is one of the most promising techniques. A prospective Phase 1, open-label, single-arm clinical trial provided preliminary evidence supporting the use of MRgFUS to enhance brain penetration of trastuzumab in patients with HER2BC with brain metastases [254]. The treatment significantly increased drug uptake in the targeted lesions. Furthermore, trans-barrier chemical delivery and novel drug delivery pathways represent promising exploratory directions. ADCs and PDCs are currently research hotspots in the BCBM field due to their unique delivery mechanisms and potential to cross the BBB. Encouraging results have been observed in multiple early-stage clinical trials. For example, ANG1005, which actively traverses the BBB to deliver paclitaxel specifically to the intracranial TME, has demonstrated objective intracranial responses and an acceptable safety profile in a Phase II clinical study involving BCBM patients [231]. This provides a transferable design principle for multiple drug categories, including ADCs, PDCs, small molecules, and nucleic acid-based therapeutics. Meanwhile, exosomes and nanoparticle-based platforms are gaining traction as natural or biomimetic carriers for BBB-penetrating delivery. Their feasibility can be further enhanced by combining them with FUS-induced barrier opening to improve intralesional drug exposure.

Based on the mechanisms and insights of cellular crosstalk within the BCBM microenvironment, novel strategies for clinical translation are emerging. After crossing the BBB, some breast cancer cells undergo cell death, while the majority enter a dormant state. These dormant cells can later be “reawakened” under specific signaling cues and mechanical microenvironments. Clinically, targeting the ERK/p38 balance to induce and maintain tumor cell dormancy may serve as a promising therapeutic approach to delay or prevent the development of macrometastases [121, 255].

Current evidence from single-cell and spatial profiling studies on adaptive immunity reveals that the immunosuppressive core of BCBM is not always dominated by the PD-1/PD-L1 axis. CD8^+^ T cells exhibit an exhausted phenotype with poor infiltration, while immunosuppressive populations such as FOXP3^+^ Tregs and M2-like macrophages are markedly enriched [130, 131]. Moreover, alternative immune checkpoint pathways such as LAG-3–Galectin-3 are highly active. These findings suggest that clinical translation strategies should shift from PD-1 monotherapy toward stratified combination therapies. In BCBM, prioritizing the evaluation of combined blockade targeting LAG3 and galectin-3 and together with PD-1, alongside concurrent de-suppressive interventions against Tregs and metabolic inhibitory pathways, may hold greater immediate clinical relevance. For instance, arginine depletion mediated by ARG2 is closely linked to T cell deficiency. Combining arginine pathway modulation with checkpoint inhibition may restore the proliferative and cytotoxic capacity of effector T cells.

Therapeutic strategies targeting BAMs should focus on inhibiting their pro-metastatic functions. BAMs enhance BBB permeability through the secretion of various factors; this process may be blocked using anti-VEGF antibodies, such as bevacizumab, or MMP inhibitors. Additionally, BAM-secreted GDNF promotes cancer cell survival in the leptomeninges, suggesting that inhibitors of the GDNF signaling pathway, such as RET inhibitors, could improve treatment outcomes. For neutrophils, rather than simply depleting them, reprogramming N2 neutrophils toward the N1 anti-tumor phenotype via IFN-β or TGF-β blockade may better align with safety requirements in the CNS. Furthermore, targeting neutrophil recruitment pathways, such as using CXCR2 antagonists to inhibit the CXCL2–CXCR2 axis, could reduce their migration into the brain. MDSCs suppress NK cell activity and promote tumor progression via signaling pathways involving TGF-β and JAK-STAT5. Thus, inhibitors targeting these pathways may enhance the effector functions of anti-tumor immune cells. Strategies to counteract NK cell suppression should aim to restore their cytotoxicity. Since NK cell function in the brain is often impaired by hypoxia, adenosine, and inhibitory receptors, combinations such as IL-15 super-agonists, A2A receptor antagonists, and anti-NKG2A antibodies may help restore their anti-tumor activity.

Astrocytes promote BCBM through physical support, secretion of soluble factors, and immunosuppression. Therapeutic strategies should therefore focus primarily on reversing their immunoinhibitory functions. Developing Cdk5 inhibitors or blocking downstream Nlrc5 signaling may alleviate astrocyte-mediated suppression of T cells. Additionally, neutralizing pro-metastatic factors secreted by astrocytes, such as CHI3L1 and TNFα, and disrupting their physical support represent promising research directions. Another key strategy involves modulating the polarization state of microglia. Promoting M1 polarization via TLR agonists, such as LPS analogs, while inhibiting M2 polarization by blocking IL-4, IL-10, or TGF-β signaling, are viable approaches. Tamoxifen has been shown to enhance M1 polarization and phagocytic capacity of microglia, however, its delivery into the CNS requires optimized strategies such as nanocarriers or intrathecal administration. Furthermore, given that M2-polarized microglia upregulate PD-L1, combining PD-1/PD-L1 inhibitors with M1-polarizing agents may yield synergistic antitumor effects. Targeting strategies for bmCAFs should aim to counteract their roles in matrix remodeling and metabolic immunosuppression. Inhibiting fucosylation pathways may reduce bmCAF-promoted tumor migration and invasion. Moreover, neutralizing antibodies against pro-inflammatory cytokines secreted by bmCAFs, such as IL-6, could be beneficial. The combination of these approaches with immune checkpoint inhibitors or adoptive T cell therapy warrants further investigation.

Looking ahead, the treatment of BCBM is poised to evolve toward multidimensional, precise, and combination-based strategies. First, the integration of AI and multi-omics technologies will enable the development of patient stratification tools and companion diagnostics. These advances will allow precise assessment of EMT status, expression of ECM components, and features of the immune microenvironment, thereby informing patient selection and combination strategy design in clinical trials. Second, innovative approaches that combine physical modalities, novel drug formats, and nanocarrier technologies will be essential for the dual modulation of the BBB. Furthermore, immunotherapy must look beyond the PD-1/PD-L1 axis alone and explore blockade of alternative immune checkpoints, such as LAG-3, to remodel the antitumor immune landscape within the brain. In addition, therapeutic targeting of specific cellular components, including astrocytes, microglia, BAMs, neutrophils, and MDSCs, should emphasize dynamic polarization and fine-tuned functional modulation rather than mere depletion. This approach will help balance treatment efficacy with safety within the CNS. In Table 6, we list investigational drugs targeting EMT, ECM remodeling, angiogenesis, the BBB, and immune-based strategies, whose clinical trial efficacy data are expected to provide critical guidance for the treatment of BCBM. Finally, interdisciplinary collaboration, innovative clinical trial designs, and multi-level drug development will be critical to translating these strategies into clinical practice, ultimately bringing truly precision medicine breakthroughs to patients with BCBM.

**Table 6:** Investigational agents targeting key processes in breast cancer brain metastases

Drug/Intervention	Mechanism of action	Target/Pathway	Trial ID	Phase	Patient Population	Intervention	Status	Outcome/Supporting evidence	References
Fresolimumab (GC1008)	pan-TGF-β neutralizing antibody	TGF-β/SMAD pathway	NCT01401062	Phase 2	metastatic breast cancer, including brain metastasis (BM)	Fresolimumab+radiotherapy	Completed	a better overall survival of patients received a higher dose of resolimumab	[256]
Galunisertib (LY2157299)	next-generation TGF-βR1 inhibitor	TGF-β/SMAD pathway	NCT02160106	Phase 1b	triple negative metastatic breast cancer, including BM	Galunisertib+paclitaxel	Completed	No results posted yet	/
Vantictumab (OMP-18R5)	frizzled receptor mAb	Wnt/β-catenin pathway	NCT01973309	Phase 1b	HER2-negative metastatic breast cancer, including BM	Vantictumab+paclitaxel	Completed	Acceptable safety; 6-gene WNT pathway signature showed significant association with progression-free survival (PFS) and overall survival (OS).	[257]
AL101	Notch inhibitor	Notch signaling pathways	NCT04461600	Phase 2	Notch-activated recurrent or metastatic TNBC, including BM	AL101	Closed for enrollment	No results posted yet	[258]
Ipatasertib	AKT inhibitor	PI3K/AKT/mTOR pathway	NCT03853707	Phase 1/1b	metastatic breast cancer, including brain metastasis (BM)	Ipatasertib+carboplatin/paclitaxel	Completed	ORR = 20%; PFS = 4.8 months;	[259]
Bevacizumab	anti-VEGF mAb	Angiogenesis	NCT01004172	Phase 2	BCBM	Bevacizumab +carboplatin	Completed	CNS-ORR = 63%; median PFS = 5.62 months; median OS = 14.1 months	[260]
Bevacizumab	anti-VEGF mAb	Angiogenesis	NCT02185352	Phase 2	BCBM	Bevacizumab +etoposide+cisplatin +WBRT	Completed	median brain-specific PFS = 8.1 months; 8-months brain-PFS = 48.7%; median OS = 15.9 months; 2-month brain-specific ORR = 41.9%	[261]
Bevacizumab	anti-VEGF mAb	Angiogenesis	NCT05357417	Phase 2	ERBB2-negative mBC with active BMs	Bevacizumab +utidelone	Completed	CNS ORR = 42.6%; median PFS = 7.7 months; median CNS PFS = 10.6 months; median OS = 15.1 months	[262]
Apatinib	VEGFR-2 TKI	Angiogenesis	/	Observational reported (not RCT)	HER2-negative breast cancer with BMs	Apatinib+chemotherapy	/	CNS ORR = 17.2%;CBR = 53.4%; median CNS PFS = 6.4 months; median OS = 10.7 months	[263]
Anlotinib	Multi-target TKI (VEGFR/FGFR/PDGFR, etc.)	Angiogenesis	/	Observational reported (not RCT)	TNBC with BMs	Anlotinib	/	median CNS PFS = 7.2 months; median OS = 10.2 months; intracranial ORR = 31.0%; intracranial disease control rate (iDCR) = 86.2%	[264]
Andecaliximab (GS-5745/ADX)	anti-MMP9-mAb	ECM remodeling	NCT01803282	Phase 1	metastatic breast cancer	Andecaliximab+paclitaxel	Completed	median PFS = 7.4 months; ORR = 53%; CR = 7%.	[265]
Roneparstat (SST0001)	Heparanase inhibitor	ECM remodeling	NCT01764880	Phase 1	Advanced multiple myeloma	Roneparstat (SST0001)	Completed	PR = 5.9%; SD = 52.9%. In BCBM, HPSE promotes ECM and BBB degradation and therapy resistance; BCBM models show HPSE drives lapatinib resistance and brain colonization; HPSE inhibitors restore sensitivity	[266]
AD-5584, AD-8007	Brain-penetrant ACSS2 inhibitors	Metabolic reprogramming	/	Preclinical (BCBM models)	BCBM mouse models	AD-5584, AD-8007	/	reducing pre-formed tumors; blocking BCBM tumor growth; reducing tumor burden and extending survival	[267]
MRgFUS	Open BBB	Open BBB	NCT03714243	Phase 1	HER2BC with BMs	MRgFUS+trastuzumab	Completed	average standardized uptake value ratio (SUVR) = 101 ± 71%; enhanced drug uptake in 87 ± 17% of sonicated voxels; unidimensional tumor measurements decreased by 19 ± 12%	[254]
Intra-arterial mannitol BBB disruption	Open BBB	Open BBB	NCT00397501	Phase 1/2	BCBM	Mannitol BBBD + methotrexate/carboplatin	not yet recruiting	No results posted yet	[268]
Pembrolizumab	PD-1 inhibitor	PD-1, reversing T-cell exhaustion	NCT02886585	Phase 2	Leptomeningeal metastasis (major breast cancer)	Pembrolizumab (intravenous injection)	Completed	60% patients were alive at 3 months	[269]
Pembrolizumab	PD-1 inhibitor	PD-1	NCT03449238	Phase 1b/2	BCBM	Pembrolizumab+stereotactic radiosurgery (SRS)	Ongoing	/	[270]
Pembrolizumab	PD-1 inhibitor	PD-1	NCT02886585	Phase 2	BMs, including BCBM	Pembrolizumab	Completed	Intracranial benefit rate = 42.1%	[238]
Nivolumab	PD-1 inhibitor	PD-1	NCT03807765	Phase 1b	BCBM	Nivolumab+SRS	Completed	Median intracranial control = 6.2 months; 6- and 12-month control rates = 55% and 22%	[271]
Atezolizumab	PD-L1 inhibitor	PD-L1	NCT03483012	Phase 2	TNBC with BMs	Atezolizumab+SRS	Ongoing	/	[272]
HER2 CAR-T	HER2-CAR T cells	T cells	NCT03696030	Phase 1	Active brain and/or leptomeningeal metastases, including breast cancer)	HER2-CAR T cells (regional/intrathecal/locoregional)	Ongoing	/	[273]
Relatlimab + nivolumab	PD-1 inhibitor+LAG-3 inhibitor	LAG-3+PD-1	NCT05704647	Phase 2	Active melanoma brain metastases	Relatlimab + nivolumab	Ongoing	/	[274]
LAG525+spartalizumab	PD-1 inhibitor+LAG-3 inhibitor	LAG-3+PD-1	NCT03499899	Phase 2	advanced/metastatic TNBC	LAG525+spartalizumab	Completed	ORR = 5%	[275]
LAG525+spartalizumab+carboplatin	PD-1 inhibitor+LAG-3 inhibitor+chemotherapy	LAG-3+PD-1+chemotherapy	NCT03499899	Phase 2	advanced/metastatic TNBC	LAG525+spartalizumab+carboplatin	Completed	ORR = 32.4%	[275]
LAG525+carboplatin	LAG-3 inhibitor+chemotherapy	LAG-3+chemotherapy	NCT03499899	Phase 2	advanced/metastatic TNBC	LAG525+carboplatin	Completed	ORR = 17.6%	[275]
GB1211+atezolizumab	Galectin-3 inhibitor+PD-L1 inhibitor	Galectin-3+PD-L1	GALLANT-1 (NCT05240131	Phase 1b/2a	NSCLC	GB1211(oral)+atezolizumab	Ongoing	/	[276]
Tiragolumab + atezolizumab	TIGIT inhibitor+PD-L1 inhibitor	TIGIT+PD-L1	NCT06175390	Phase 2	TNBC	Tiragolumab+ atezolizumab±chemotherapy	Recruiting	/	[277]
BLZ945	CSF1R inhibitor	CSF1R controls BAM survival and polarization	/	preclinical models	TNBC	BLZ945	/	reduced the formation of brain metastases in both models by 57–65%; reduced the number and size of metastases in both models by 44–72%	[278]
CAR-NK	CAR-NK	NK cell replacement or boosting	NCT03383978	Phase 1	Glioblastoma	CAR-NK (intracranial injection)	Recruiting	/	[279]
Leronlimab (PRO 140)	CCR5 blockade	Blocking neutrophils or MDSCs recruitment	NCT04313075	Phase 1b/2	metastatic TNBC	Leronlimab (PRO 140)	Recruiting	increase OS and PFS	[280]
Bemcentinib (BGB324)	AXL inhibitor	GAS6–AXL signaling (bmCAF–tumor crosstalk)	NCT03184558	Phase 2	TNBC	BGB324+pembrolizumab	Completed	Unpublished	[281]
Tocilizumab	anti-IL6 mAb	IL-6 R blockade (bmCAFs-astrocyte IL-6 axis)	NCT05846789	Phase 2	metastatic TNBC	Tocilizumab+SOC chemotherapy	Recruiting	/	[282]a

### The integration of modern technology in the clinical diagnosis and treatment of BCBM

**Fig. 6:**
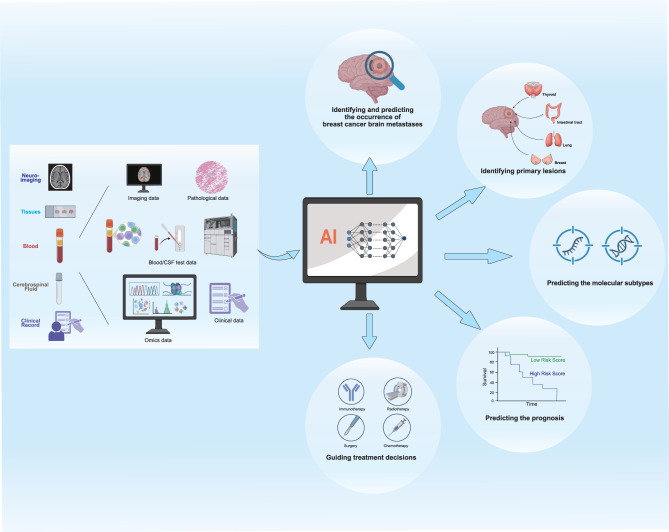
Application of modern technologies in breast cancer brain metastases clinical diagnosis and treatment. In the era of artificial intelligence (AI), the rapid advancement of modern technologies including imaging, proteomics, and metabolomics has significantly propelled the diagnosis and treatment of breast cancer brain metastasis (BCBM). A diverse array of data, encompassing imaging data, histopathological data, multi-omics data, and clinical data, is processed by AI to aid in the identification and prediction of BCBM occurrence, localization of primary lesions, prediction of molecular subtypes, prognosis of patient outcomes, and guidance of treatment decisions

In the era of AI, the rapid advancement of modern technologies such as imaging omics, proteomics, metabolomics, and others has significantly propelled the diagnosis and treatment of BCBM (Fig. 6). The successful application of AI in cancer diagnosis, treatment, and scientific research has unlocked great potential for the evolution of precision medicine.

The combination of AI and radiomics has proven instrumental in predicting the occurrence, identifying primary lesions, and forecasting molecular subtypes of BCBM. Multiple risk prediction models have been developed to estimate the likelihood of BCBM, thereby assisting clinicians in implementing targeted prevention strategies. For instance, a study by Wu et al. introduced a predictive model that integrates 20 clinicopathological features and employs Fine-Gray competing risk regression analysis to forecast the risk of brain metastases in breast cancer patients [283]. This model demonstrated a C-index of less than 0.70 and area under the curve (AUC) values of 0.674, 0.670, and 0.729 for 1-year, 3-year, and 5-year BCBM risk predictions, respectively. This predictive model demonstrates strong clinical applicability and offers valuable support for assisting clinical decision-making.

In addition, deep learning models can identify primary lesions associated with brain metastases by analyzing differences in their anatomical distribution. For instance, Mahmoodifar et al. employed deep learning and machine learning algorithms to distinguish the distribution patterns of brain metastases originating from different primary cancers [284]. Their study achieved high accuracy rates of 96–97% by comparing the anatomical spread patterns between brain metastases from breast cancer versus lung cancer and from breast cancer versus kidney cancer. This capability enables clinicians to pinpoint the primary lesion by examining the brain metastases, thereby facilitating the implementation of appropriate treatment measures.

AI methods have also been utilized to predict the molecular subtypes of brain metastases derived from breast cancer. Given that a significant proportion of breast cancer patients exhibit receptor discrepancies between the primary tumor and brain metastases, treatment decisions are considerably impacted. Consequently, multiple models have been developed to non-invasively predict the receptor status of BCBM by constructing radiomic signatures that analyze the receptor status of both the primary breast cancer and corresponding brain metastases. For instance, Luo et al. employed preoperative brain MRI to develop machine learning-based radiomic signatures that predict the status of ER, PR, and HER2 in BCBM with accuracies of 0.89, 0.88, and 0.87, respectively [285]. Similarly, Young et al. constructed a model that predicted HER2 status with a high accuracy of 0.98, based on relative cerebral blood volume [286].

In addition, AI has the potential to predict the prognosis of patients with brain metastases and guide treatment decisions. For instance, Li et al. constructed an XGBoost model to predict the survival rate of patients with BCBM. The model demonstrated strong performance, with survival AUC values greater than 0.8 from 6 months to 3 years, indicating its robust predictive capability [287]. The deep learning-based dose optimization method for BCBM SRS, developed by Pandey et al., leverages multiparametric MRI images to construct a model [288]. This innovative approach not only helps optimize the SRS dose but also achieves precise targeting of BCBM while effectively avoiding toxic side effects such as radionecrosis.

Systematic reviews and meta-analyses provide an evidence-based foundation for the application of AI in medicine. Recent quantitative evidence indicates that deep learning approaches have achieved high overall performance in the detection and segmentation of brain metastases using MRI. A meta-analysis including only deep learning-based studies demonstrated that these algorithms can effectively detect and segment brain metastases in MRI images, with patient-level and lesion-level sensitivity reaching 86% and 87%, respectively [289]. Although there are currently limited systematic reviews specifically focused on AI in BCBM, a growing body of research highlights its potential value in clinical applications for BCBM. While AI has exhibited tremendous potential in multiple domains of BCBM research, it also confronts numerous challenges. On one hand, obtaining large-sample, high-quality data is a prerequisite for the smooth application of AI. The performance of AI models heavily relies on large-scale, high-quality datasets with consistent annotations for training and validation [290, 291]. As the significance of multimodal fusion analysis becomes increasingly prominent, the demand for diverse and standardized data has further intensified. Currently, retrospective data are often used as the primary source for model development due to their accessibility; however, variations in imaging acquisition equipment, scanning parameters, and clinical protocols across different centers and studies introduce substantial heterogeneity and bias into such data, severely undermining the models’ generalizability and the reproducibility of their results. In contrast, RCTs can provide rigorously standardized, high-quality data, but they are associated with high costs, long durations, and considerable implementation difficulties. Therefore, to advance the robust development of AI in the field of BCBM research, it is imperative to strengthen multi-center, cross-institutional collaboration and establish data-sharing mechanisms. Integrating data resources from various institutions to construct large-scale, high-quality, and standardized public datasets will significantly enhance the reliability and diversity of model training and validation. In recent years, multicenter public challenges and large-scale datasets have provided a solid foundation for reproducible model evaluation and cross-institutional external validation. For instance, an analysis of the National Cancer Database (NCDB) encompassing 2,610,598 breast cancer cases recorded between 2010 and 2020 included 9,005 patients with de novo BCBM. This study delineated associations among various treatment strategies, molecular subtypes, and survival outcomes, offering a robust baseline for the external validation of clinical endpoints in BCBM risk and prognostic models. A population-based registry study from Ontario, Canada, identified patients diagnosed with de novo BCBM between 2009 and 2018. The study quantified the cumulative incidence of BCBM in relation to breast cancer molecular subtypes. These findings provide valuable real-world evidence for calibrating AI-driven risk stratification models and evaluating subgroup disparities [292]. Meanwhile, interdisciplinary collaboration is indispensable in this process. AI research on BCBM requires the integration of expertise from fields such as oncology, radiology, pathology, and computational biology. Although differences in terminology systems and research paradigms across disciplines may pose certain challenges to collaboration, such in-depth integration remains a key driver for bridging the gap from data to clinical application. On the other hand, the interpretability and generalizability of models represent two core challenges for the clinical application of AI. Deep learning models are often regarded as “black boxes” due to the lack of transparency in their decision-making processes; end-users struggle to understand how input data are mapped to output results, and this issue is particularly pronounced in trust-sensitive medical scenarios. Thus, improving model interpretability is not only a technical necessity but also a prerequisite for clinical acceptance. By incorporating explainable AI (XAI) methods, such as feature importance analysis, attention mechanisms, and visualization tools, the internal operational logic of models can be unveiled, enhancing the transparency and credibility of their decision-making processes [293, 294]. The generalizability of models is also of crucial importance. Differences in imaging equipment, scanning protocols, and patient demographics across medical institutions often lead to a significant decline in model performance during cross-institutional and cross-regional validation. To improve the adaptability of models to diverse populations and tumor types, it is necessary to construct more diverse datasets through multi-center collaboration and adopt strategies such as domain adaptation, adversarial training, and structural optimization to enhance model robustness. These approaches help improve the performance of models on unseen data and facilitate their transition from experimental settings to real-world clinical applications.

## Conclusions and future prospects

BCBM is a major factor contributing to the sharp decline in survival among breast cancer patients. The initiation and progression of BCBM involve a complex, multi-step process that includes both the intrinsic evolution of tumor cells and the remodeling of the unique brain microenvironment. This review provides a systematic illustration of the core mechanisms underlying BCBM, from EMT, ECM remodeling, metabolic reprogramming to BBB breaching, demonstrating how cancer cells adapt to and exploit this distinct ecological niche. More importantly, we offer an in-depth analysis of the highly coordinated immunosuppressive network within the BCBM TME. This includes the dysfunction of adaptive immune cells, such as T cells and B cells, the remarkable plasticity and pro-metastatic functions of innate immune cells, including macrophages, neutrophils, NK cells, and MDSCs, as well as the pivotal roles played by CNS-specific components, like astrocytes, microglia, and bmCAFs, in shaping an immune-privileged niche. Together, these mechanisms form the cornerstone of treatment resistance in BCBM.

Despite significant advances in molecular subtype-guided systemic therapies, including anti-HER2 agents, CDK4/6 inhibitors, and ADCs, as well as local treatments such as radiotherapy and surgery, the efficacy of these interventions remains substantially limited in BCBM. Major obstacles include the BBB, inadequate intracranial drug delivery, a highly immunosuppressive TME, and profound tumor heterogeneity. Moving forward, overcoming BCBM will require a paradigm shift toward multidimensional, precision-guided, and combinatory therapeutic strategies. First, there is an urgent need to develop mechanism-driven combination therapies that concurrently target multiple cellular populations and signaling pathways to achieve synergistic antitumor effects. Second, innovative drug delivery platforms, such as BBB-penetrating compounds, focused ultrasound, and nanocarrier systems, must be leveraged to enhance drug concentrations in the brain. Meanwhile, the integration of AI and multi-omics data is critical to building predictive models for improved patient stratification and personalized treatment selection. Furthermore, innovative clinical trial designs incorporating CNS-specific endpoints should be implemented to accelerate the translation of effective therapies into clinical practice. In conclusion, a deeper understanding of the molecular and cellular mechanisms underlying BCBM has unveiled a spectrum of potential therapeutic targets. Future breakthroughs will rely on interdisciplinary collaboration that bridges fundamental research and clinical applications, harnesses cutting-edge technologies for drug development, and ultimately transforms the treatment landscape, offering renewed hope for patients with BCBM.

## Data Availability

No datasets were generated or analysed during the current study.

## Abbreviations

TME: Tumor microenvironment
BCBM: Breast cancer brain metastasis, breast cancer brain metastases
HR: Hormone receptors
HER2: Human epidermal growth factor receptor 2
ER: Estrogen receptor
PR: Progesterone receptor
CNS: Central nervous system
BBB: Blood-brain barrier
BCFB: Blood-cerebrospinal fluid barrier
EMT: Epithelial-mesenchymal transition
MMPs: Matrix metalloproteinases
BMECs: Brain microvascular endothelial cells
VEGF: Vascular endothelial growth factor
TCA: Tricarboxylic acid
Tregs: Regulatory T cells
TILs: Tumor-infiltrating lymphocytes
TLS: Tertiary lymphoid structures
BAMs: Border-associated macrophages
GDNF: Glia-derived neurotrophic factor
TANs: Tumor-associated neutrophils
ECM: Extracellular matrix
MDSCs: Myeloid-derived suppressor cells
CAFs: Cancer-associated fibroblasts
LPS: Lipopolysaccharide
INF -γ: Interferon-γ
TNF-α: Tumor necrosis factor-α
bmCAFs: Brain metastatic cancer-associated fibroblasts
CTCs: Circulating tumor cells
ctDNA: Circulating tumor DNA
CEA: Carcinoembryonic antigen
CA153: Carbohydrate antigen 153
POSTN: Periostin
SRS: Stereotactic radiosurgery
TKIs: Tyrosine kinase inhibitors
SD: Disease stabilization
CDK4/6: Cyclin-dependent kinases 4 and 6
PD-1: Programmed death receptor-1
CTLA-4: Cytotoxic T lymphocyte antigen-4
ADCs: Antibody-drug conjugates
PDCs: Peptide-drug conjugates
PARP: Poly (ADP-ribose) polymerase
AI: Artificial intelligence
AUC: Area under the curve
OS: Overall survival
PFS: Progression free survival
ORR: Objective response rate
